# Designing reliable and accurate isotope-tracer experiments for CO_2_ photoreduction

**DOI:** 10.1038/s41467-023-38052-0

**Published:** 2023-05-03

**Authors:** Shengyao Wang, Bo Jiang, Joel Henzie, Feiyan Xu, Chengyuan Liu, Xianguang Meng, Sirong Zou, Hui Song, Yang Pan, Hexing Li, Jiaguo Yu, Hao Chen, Jinhua Ye

**Affiliations:** 1 College of Science, Shenzhen Institute of Nutrition and Health, Huazhong Agricultural University, 430070 Wuhan, P. R. China; 2 International Center for Materials Nanoarchitectonics (WPI-MANA), National Institute for Materials Science (NIMS), 1-1 Namiki, Tsukuba, Ibaraki, 305-0044 Japan; 3 Shenzhen Branch, Guangdong Laboratory for Lingnan Modern Agriculture, Genome Analysis Laboratory of the Ministry of Agriculture, Agricultural Genomics Institute at Shenzhen, Chinese Academy of Agricultural Sciences, 518120 Shenzhen, P. R. China; 4 The Education Ministry Key Lab of Resource Chemistry, Joint International Research Laboratory of Resource Chemistry, Shanghai Frontiers Science Center of Biomimetic Catalysis, College of Chemistry and Materials Science, Shanghai Normal University, 200234 Shanghai, China; 5 Laboratory of Solar Fuel, Faculty of Materials Science and Chemistry, China University of Geosciences, 430074 Wuhan, P. R. China; 6 National Synchrotron Radiation Laboratory, University of Science and Technology of China, 230029 Hefei, P. R. China; 7 Hebei Provincial Key Laboratory of Inorganic Nonmetallic Materials, College of Materials Science and Engineering, North China University of Science and Technology, 063210 Tangshan, P. R. China; 8 Graduates School of Chemical Science and Engineering, Hokkaido University, Sapporo, 060-0814 Japan; 9 TU-NIMS International Collaboration Laboratory, Tianjin University, 300072 Tianjin, P. R. China

**Keywords:** Photocatalysis, Mass spectrometry, Artificial photosynthesis, Photochemistry

## Abstract

The photoreduction of carbon dioxide (CO_2_) into renewable synthetic fuels is an attractive approach for generating alternative energy feedstocks that may compete with and eventually displace fossil fuels. However, it is challenging to accurately trace the products of CO_2_ photoreduction on account of the poor conversion efficiency of these reactions and the imperceptible introduced carbon contamination. Isotope-tracing experiments have been used to solve this problem, but they frequently yield false-positive results because of improper experimental execution and, in some cases, insufficient rigor. Thus, it is imperative that accurate and effective strategies for evaluating various potential products of CO_2_ photoreduction are developed for the field. Herein, we experimentally demonstrate that the contemporary approach toward isotope-tracing experiments in CO_2_ photoreduction is not necessarily rigorous. Several examples of where pitfalls and misunderstandings arise, consequently making isotope product traceability difficult, are demonstrated. Further, we develop and describe standard guidelines for isotope-tracing experiments in CO_2_ photoreduction reactions and then verify the procedure using some reported photoreduction systems.

## Abstract (web summary)

Current verification of CO_2_ photoreduction products using isotope-tracer methods is insufficient and can provide false-positive results. Here, rigorous protocols and case studies are presented to avoid pitfalls in isotope-tracer experiments.

## Introduction

If sunlight can be efficiently harnessed as a primary energy source for the photoreduction of CO_2_ into synthetic fuels, it will contribute a large flow of carbon-neutral energy in a material format that is compatible with existing fossil fuel infrastructure^1–8^. At a large enough scale, this technology may enable the control of atmospheric CO_2_ levels and make fossil fuels obsolete in the coming decades^9–12^. However, considerable scientific and technical challenges must be overcome to realize CO_2_ photoreduction reactions with high selectivity and conversion efficiency^13–18^. After many decades of exploration, most experimental studies focus on optimizing materials and reaction systems^19–24^. Most reported CO_2_ photoreduction systems are not yet able to deliver the desired products on a practical scale and with long-term operational stability. The low selectivity and CO_2_ conversion efficiency of most existing reaction systems^25^ is a significant roadblock in this field because it is challenging to attribute potential products (e.g., CO, alkanes, alcohols, carboxylic acids, and alkenes) solely to CO_2_ photoreduction processes^26,27^. Unfortunately, the efficiency of some high-performance reaction systems is later attributed to the decomposition of the carbon contaminants on the photocatalyst or the reaction system^28–32^.

Increasing awareness of the challenge of attributing products to CO_2_ photoreduction has made ^13^C isotope labeling experiments essential^33–35^. Lehn et al. carried out isotope labeling experiments with ^13^CO_2_ in 1983, revealing that the reduction product of CO indeed came from CO_2_ by using gas chromatography-mass spectrometry (GC-MS). However, no standard test method was mentioned in this research^36,37^. Later, Willner et al. used ^13^C nuclear magnetic resonance spectroscopy (NMR) to show that H^13^COO^−^ originates from H^13^CO_3_^−^ (the dissolved ^13^CO_2_ in solution)^38^. Over the years, various techniques using NMR spectroscopy and Fourier transform infrared spectroscopy (FT-IR) have been employed in isotope-tracer studies: the approach of ^1^H-NMR is efficient for liquid product analysis through peak shifts and coupling constants of the ^13^C-linked hydrogen; FT-IR equipped with a gas cell is effective for gaseous product analysis via the increased path length of a beam by multiple internal reflections^39,40^. Still, both methods are relatively insensitive to various carbon isotopes. Thus GC-MS-based techniques are still the most reliable and universal strategy for isotope-tracer studies in CO_2_ photoreduction^41,42^. For example, numerous organic carbon-containing photocatalysts, such as conjugated polymers, Metal-organic frameworks (MOFs), and Covalent organic framework (COFs), have good activities in CO_2_ photoreduction^43–48^. Isotope-tracing studies with GC-MS allow researchers to exclude carbon contamination from the decomposition of these catalysts or the materials synthesis process. Current isotope-tracer methods can measure simple samples such as ^13^CO, H^13^COOH, ^13^CH_4_, ^13^C_2_H_6_, ^13^C_2_H_4_, and ^13^CH_3_OH due to the isotope effects induced by different mass-to-charge ratios^49–52^. However, once the abovementioned series of molecular substances are generated during CO_2_ photoreduction, the inherent ionization process of mass detection could not only charge the molecules but also inevitably cause chemical bonds to break in these molecules, making the isotope-tracing via mass detection becomes increasingly difficult as molecular fragments with similar *m*/*z* ratios interfere and lead to misidentification and errors in quantification. Even for the emerging synchrotron vacuum ultraviolet photoionization mass spectrometry (SVUV-PIMS) strategy, the existence of interfering factors induced by the long-time sampling is also uncertain^53^. So the gas chromatograph (GC) is essential to be added to the sampling system to separate molecular species before collecting mass spectra (MS)^54,55^.

Despite the fact that isotope-tracer experiments are regarded as solid corroborating evidence for CO_2_ photoreduction, the current protocols are still substandard, causing the literature to be rife with false-positive results^56^. Meanwhile, the accurate and effective solution of isotope detection for various products in CO_2_ photoreduction is still lacking, which causes the community to be suspicious of all results. In the present work, we confirmed the pros and cons of the current mass spectrometry protocol as well as the emerging technology of synchrotron vacuum ultraviolet photoionization mass spectrometry (SVUV-PIMS) when they applied to the isotope-tracer experiments in CO_2_ photoreduction. We also experimentally performed isotope-tracer experiments on various isotope standards and described a standard protocol assessing various potential products of CO_2_ reduction. In addition, some classic CO_2_ photoreduction systems reported in the literature were also used to validate our protocols. It is imperative to settle on an appropriate scientific method for isotope-tracer studies to promote trust in the community as we develop and benchmark some materials systems for CO_2_ photoreduction. This research illustrates the pitfalls and misunderstandings in isotope-tracer studies and also provides examples and references for ^13^C isotope-tracer methods so we can all obtain solid evidence that reduction products are firmly attributed to CO_2_ photoreduction reactions.

## Results

### Importance of separation for multicomponent samples

**Fig. 1:**
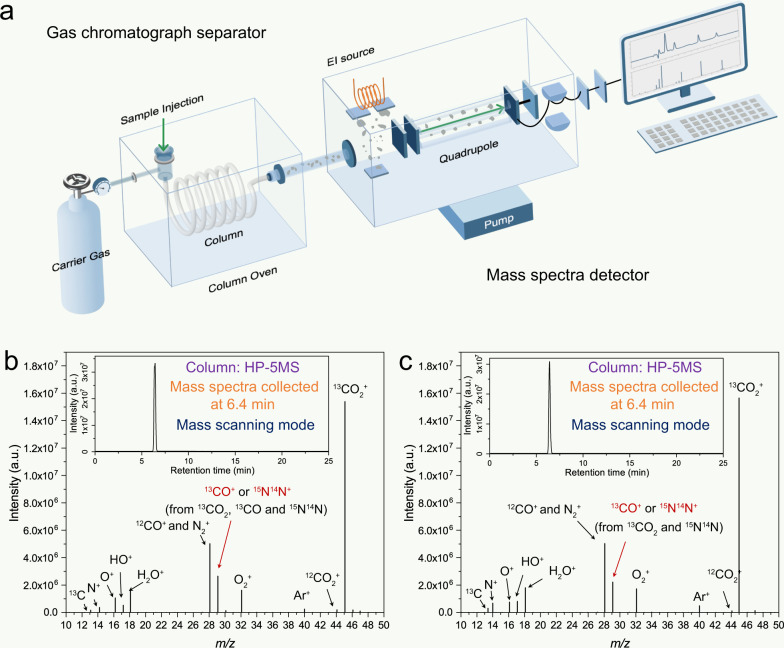
The contemporary approach toward isotope-tracing experiments in CO_2_ photoreduction. **a** An illustration of a GC-MS with the GC separator and MS detector used to trace isotopes in CO_2_ reduction. **b**, **c** MS spectra and TIC (inset) using an HP-5ms column for the model gases collected from the sealed CO_2_ reduction system: **b** after photoreduction (^13^CO_2_, ^13^CO, and vapor) and **c** before photoreduction (^13^CO_2_ and vapor). Source data are provided as a Source Data file.

GC-MS is the most common instrument for tracing isotopes in CO_2_ reduction reactions currently. The GC-MS instrument is composed of a GC to separate molecular species based on affinity with a column material and an MS to detect their mass or their mass fragments (Fig. 1a). However, the current method for tracing isotopes in CO_2_ reduction reactions ignores this. We verified the reliability of the current method. To mimic the products and conditions of a real photoreduction reaction, we directly employed standard gases such as ^13^CO_2_, ^13^CO, and vapor in the CO_2_ reduction system. The volume ratio of ^13^CO >5% v/v is much higher than the yield of CO in the usual CO_2_ photoreduction process. Then these premixed gases were collected from the CO_2_ reduction system and injected into the GC-MS equipped with a commercial HP-5ms column (see “Methods” for experimental details). As shown in Fig. 1b, a peak at *m/z* = 29 in the mass spectrogram could be obtained from TIC at an RT of 6.4 min. This peak has a definite value, but it could originate from three sources: (i) it is a molecular ion of ^13^CO (^13^CO^+^, *m/z* = 29), (ii) a fragment ion of ^13^CO_2_ (^13^CO^+^, *m/z* = 29), or (iii) it is a molecular ion of ^15^N^14^N (the natural abundance of nitrogen isotope, ^15^N^14^N^+^, *m/z* = 29). This could be further confirmed via the GC-MS analysis of pure ^13^CO_2_, ^13^CO, and N_2_ (Supplementary Figs. 1–3). Although the natural abundance of the nitrogen isotope exists in traces, the ^13^CO_2_ fragment ion is abundant and has the same characteristics to interfere with the isotope-tracer results for ^13^CO. Consequently, even if there is no ^13^CO in the injected mixture (only ^13^CO_2_ and vapor were injected into GC-MS), a similar mass spectrogram can be obtained under the same conditions (Fig. 1c), generating a peak at *m/z* = 29. This peak could be mainly attributed to the fragment ion (^13^CO^+^) generated from the dissociation of ^13^CO_2_. Even the deactivated fused silica tube (the length is 5 m), without any separation effect, acts as the connector to let all components (^13^CO_2_ and vapor) enter the quadrupole together; a similar result can also be obtained in the mixture of ^13^CO_2_ and vapor even without a photocatalytic reduction process (Supplementary Fig. 4). Moreover, the selected *m/z* = 45, 29 and 17 can be detected (Supplementary Fig. 5) when the detection method of GC-MS is set to the selected ion monitoring (SIM) mode (see “Methods” for experimental details). It is always used as evidence of traceability for the products originating from CO_2_ photoreduction, and signals at *m/z* = 17, 29, and 45 are assigned to CH_4_, CO, and CO_2_ molecular ions, respectively^57^. However, based on the above analysis, the presence of dissociated ^13^CO_2_ (generated fragments of ^13^CO^+^, *m/z* = 29) and vapor (generated fragments of HO^+^, *m/z* = 17) throws these conclusions of traceability into doubt.

**Fig. 2:**
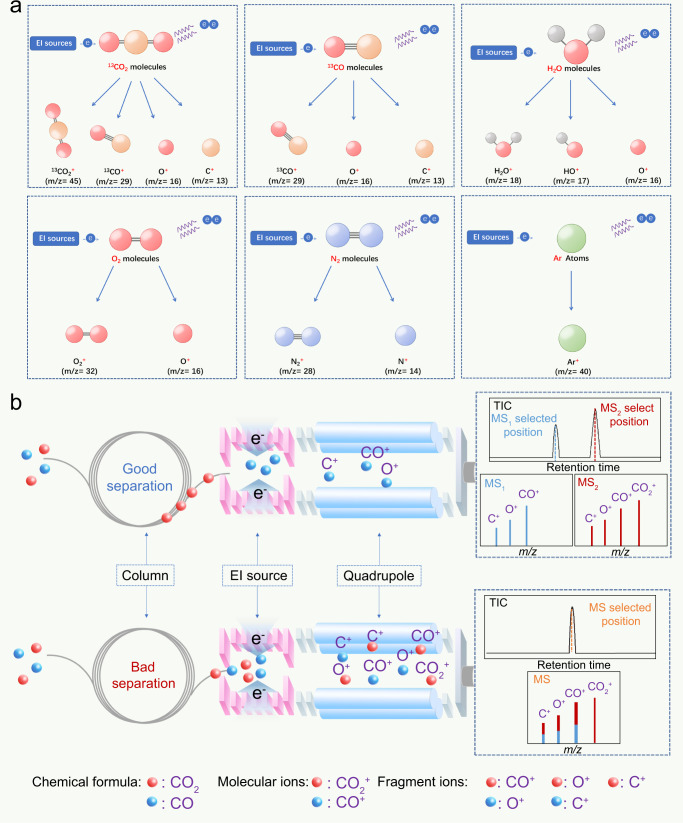
The pitfalls and misunderstandings arise in the contemporary approach. **a** An illustration of the dissociation processes as the reactants (^13^CO_2_, H_2_O), product (^13^CO), and air impurities (N_2_, O_2_, Ar) as they undergo ionization in the EI source in GC-MS. Analytical data of ^13^C isotope standard samples. **b** The illustrations of good separation condition (top) and bad separation condition (bottom) in GC for MS detection when the mixture of CO_2_ and CO to be analyzed potentially interfered with each other. Source data are provided as a Source Data file.

For such confusing results, we could ascribe them to the detection principle of MS detector for charged species. Namely, the process of charging molecules in an MS detector is not so controllable, and there is some probability that the dissociation of the molecules coincides with ionization. In particular, when the amount of the sample is large, the probabilistic dissociation and dissociative recombination become inevitable, causing molecular ions and fragmented ions with the same characteristics to interfere with each other on the MS detector^57^. As a result, confusion could occur in the isotope detection for the product of ^13^CO due to the simultaneous process of ionization and dissociation over the ^13^CO_2_ and H_2_O reactants, ^13^CO product, and air components (N_2_, O_2_, Ar), respectively (Fig. 2a).

To avoid this confusion, suitable chromatographic conditions are a prerequisite to meeting the requirement of sample separation. As shown in Fig. 2b, a GC separator equipped with a suitable chromatographic column can separate mixed gases of CO_2_ and CO so that they are ideally queued in a single file before entering the MS detector. Thus although both CO_2_ and CO can generate the same fragment ions in the ion source, their different entry times provide resolution. As a result, two signal peaks appeared at different retention times (RT) in the total ion chromatography (TIC). Their corresponding mass spectrogram (MS_1_ and MS_2_ selected at different RT) show both molecular ions and fragment ions, which can be clearly attributed to either CO or CO_2_. In contrast, chromatography columns with bad separation characteristics allow components of CO_2_ and CO to enter the ion source simultaneously and generate complex molecular ions and fragment ions. These then enter the quadrupole together, resulting in a single peak on TIC and a corresponding mass spectrogram (MS). This spectrogram is very close to the abovementioned MS_1_ obtained from the fully separated samples. However, the signal corresponding to CO^+^ could be attributed to a fragment ion of CO_2_ or a molecular ion of CO, making it impossible to distinguish the source of CO^+^. Similarly, it is impossible to attribute the signal from C^+^ or O^+^ because both CO_2_ and CO can generate it.

**Fig. 3:**
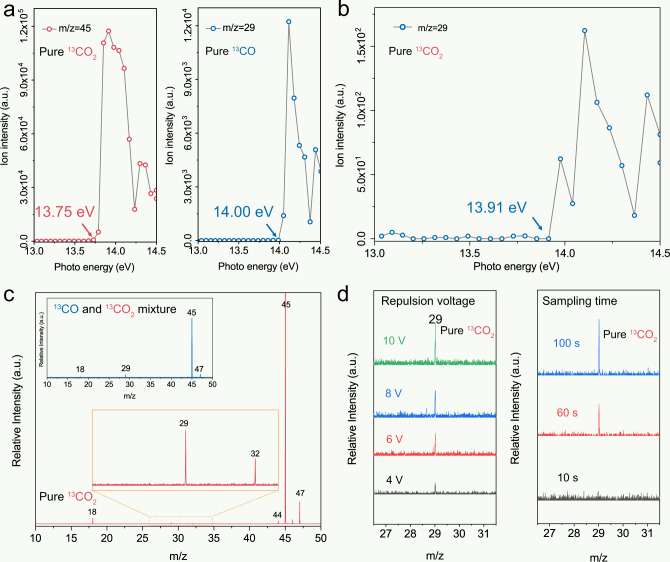
SVUV-PIMS for isotope-tracing experiments in CO_2_ photoreduction. Photoionization efficiency spectra for **a** *m*/z = 45 at a photon energy of 13.75 eV for pure ^13^CO_2_ and *m*/*z* = 29 at a photon energy of 14.00 eV for pure ^13^CO; and **b** *m*/*z* = 29 at a photon energy of 13.91 eV for pure ^13^CO_2_. SVUV-PIMS spectra of the gas components of **c** pure ^13^CO_2_ and a mixture of ^13^CO and ^13^CO_2_ (inset) at a photon energy of 14.5 eV. **d** SVUV-PIMS spectra at different repulsion voltages and sample times for *m*/*z* = 29 over pure ^13^CO_2_ at a photon energy of 14.5 eV. Source data are provided as a Source Data file.

Apart from the abovementioned method of mass spectrometry for isotope traceability, recently, an emerging technique called the synchrotron vacuum ultraviolet photoionization mass spectrometry (SVUV-PIMS) was also used to assign CO products to CO_2_ photoreduction reactions^58^. This analytical technique is useful because it delivers high-intensity, high-resolution, and widely tunable photon energies for photoionization processes. SVUV-PIMS enables selective and sensitive ionization (see “Methods” for experimental details) and can overcome the drawbacks of other ionization techniques and minimize fragmentation interference in MS detection. As shown in Fig. 3a, the *m*/*z* = 45 signal gives an ionization threshold of 13.75 eV in the photoionization spectra of pure ^13^CO_2_, while pure ^13^CO exhibits an increased ionization threshold of 14.00 eV in the *m*/*z* = 29 signal and the response rate of CO 10 times less than that of CO_2_. However, the SVUV-PIMS signal at *m*/*z* = 29 is also observed at 13.91 eV for pure ^13^CO_2_ (Fig. 3b). This feature matches the weak fragmentation of ^13^CO_2_ or via impurities introduced during the production of ^13^CO_2_ gas, interfering with the detection of ^13^CO to some extent. Therefore, when the photon energy of SVUV-PIMS was set at 14.50 eV to monitor the isotope product of ^13^CO, the peak at *m*/*z* = 29 can be found in the SVUV-PIMS spectra of pure ^13^CO_2_ (Fig. 3c) that is similar to the spectra obtained from the mixture of ^13^CO and ^13^CO_2_ (inset of Fig. 3c). Moreover, the intensity of *m*/*z* = 45 signal in pure ^13^CO_2_ is strongly associated with the increased repulsion voltage and sampling time (Fig. 3d). Although SVUV-PIMS minimizes fragmentation of the potential analytes, the popularity of this technique and the excessive demand of sample (hundred milliliters) hamper the extensive application of SVUV-PIMS to some extent. Moreover, the existence of interfering factors in this strategy induced by the long-time sampling is just like the SIM model in GC-MS, which cannot be ignored in the isotope-tracer experiment for CO_2_ photoreduction. Therefore, a complete separation of multiple components in GC is essential to obtain reliable isotope-tracing results for the products of CO_2_ photoreduction in MS.

### Analysis strategy for the isotope-labeled products of CO_2_ photoreduction

**Fig. 4:**
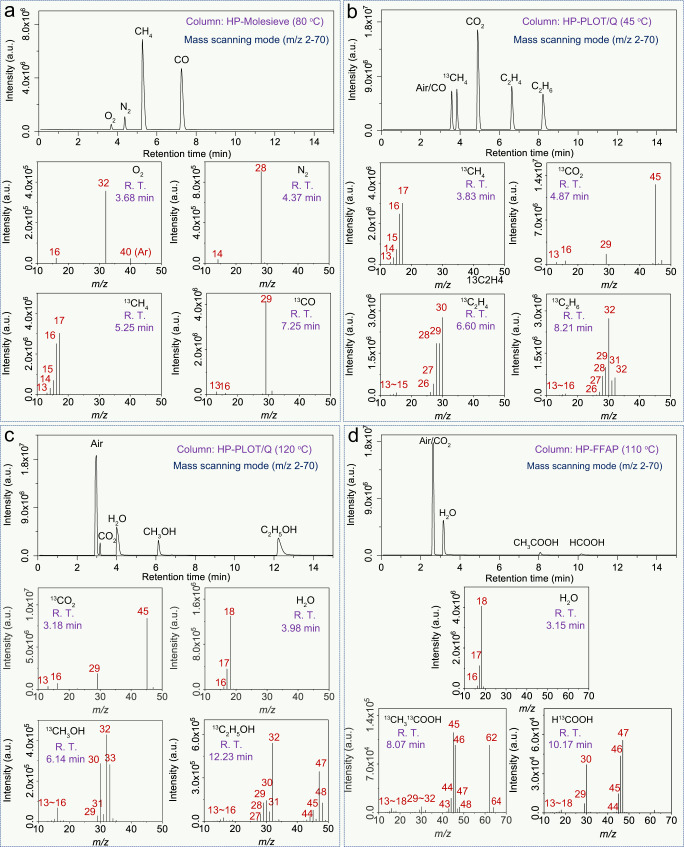
Standard guidelines for isotope-tracing experiments in CO_2_ photoreduction. Analytical data of ^13^C isotope standard samples. TIC and corresponding MS spectra for **a** O_2_, N_2_, ^13^CH_4_, and ^13^CO using the HP-Molesieve column at 80 °C; **b** ^13^CH_4_, ^13^CO_2_, ^13^C_2_H_4_, and ^13^C_2_H_6_ using HP-PLOT/Q at 45 °C; **c** ^13^CO_2_, ^13^CH_3_OH, and ^13^C_2_H_5_OH using HP-PLOT/Q at 120 °C; and **d** ^13^CO_2_, ^13^CH_3_OH, and ^13^C_2_H_5_OH using HP-FFAP at 110 °C. Source data are provided as a Source Data file.

Potential products of CO_2_ photoreduction reactions include CO, CH_4_, C_2_H_6_, C_2_H_4_, HCOOH, CH_3_COOH, CH_3_OH, and CH_3_CH_2_OH. Isotope-tracing experiments are complicated because products are inevitably mixed with the reactants (H_2_O and CO_2_) and impurities introduced during sampling (e.g., N_2_ and O_2_ via the airtight syringe). In addition, chromatographic columns such as HP-5ms in GC-MS cannot separate these gas components in isotope-tracing experiments. Thus, selecting a suitable column for separating permanent gas components is critical. For example, the HP-Molesieve column (see “Methods” for experimental details), as shown in Fig. 4a, can completely separate O_2_ (RT at 3.65 min), N_2_ (RT at 4.37 min), CH_4_ (RT at 5.25 min), and CO (RT at 7.25 min). After complete separation, it prevents the introduced air components during the injection process from interfering with the detection of products. The obtained mass spectra of O_2_ and N_2_ closely match the NIST mass spectral library (Supplementary Figs. 6 and 7), and the *m/z* of carbon-related molecular ions and fragment ions generated from ^13^CH_4_ (^13^CH_4_^+^, *m/z* = 17; ^13^CH_3_^+^, *m/z* = 16; ^13^CH_2_^+^, *m/z* = 15; ^13^CH^+^, *m/z* = 14; and ^13^C^+^, *m/z* = 13) and ^13^CO (^13^CO, *m/z* = 29 and ^13^C, *m/z* = 13) have a mass shift effect (*M*+1) compared with the non-isotope-labeled CH_4_ (Supplementary Fig. 8) and CO (Supplementary Fig. 9), respectively. Nevertheless, the molecular sieve column will irreversibly adsorb the reactant of CO_2_; thus, MS analysis of the reactant (CO_2_) and the products (CO or CH_4_) cannot be obtained simultaneously with the HP-Molesieve. To confirm the isotopic abundance of CO_2_, the HP-PLOT/Q is employed (see “Methods” for experimental details). As shown in Fig. 4b, the CH_4_ (RT at 4.87 min) and CO_2_ (RT at 6.60 min) elute as separate peaks in the TIC, and the corresponding MS of ^13^CH_4_ and ^13^CO_2_ (^13^CO_2_^+^, *m/z* = 45; ^13^CO^+^, *m/z* = 29; and ^13^C^+^, *m/z* = 13) could be obtained, respectively. The peaks exhibit a mass shift effect (*M*+1) compared to the non-isotope-labeled standards of CH_4_ (Supplementary Fig. 8) and CO_2_ (Supplementary Fig. 10). However, the air-derived components (e.g., O_2_ and N_2_) and CO (RT at 3.83 min) cannot be separated well under these conditions and may interfere with the analysis of CO isotopes. Interestingly, two kinds of C_2_ hydrocarbons (C_2_H_6_, RT at 8.21 min and C_2_H_4_, RT at 6.60 min) could be distinguished under these conditions. Although both ^13^C_2_H_4_ and ^13^C_2_H_6_ exhibit the same highest peak at *m/z* = 30, ^13^C_2_H_6_ possesses two more characteristic peaks at *m/z* = 31 and *m/z* = 32 that can only be generated from the fragment ion of ^13^C_2_H_5_^+^ and molecular ion of ^13^C_2_H_6_^+^. The ratio between the peaks that recorded in MS of ^13^C_2_H_4_ (^13^C_2_H_4_^+^, *m/z* = 30; ^13^C_2_H_3_^+^, *m/z* = 29; ^13^C_2_H_2_^+^, *m/z* = 28; ^13^C_2_H^+^, *m/z* = 27; and ^13^C_2_^+^ *m/z* = 26) and ^13^C_2_H_6_ (^13^C_2_H_6_^+^, *m/z* = 32; ^13^C_2_H_5_^+^, *m/z* = 31; ^13^C_2_H_4_^+^, *m/z* = 30; ^13^C_2_H_3_^+^, *m/z* = 29; ^13^C_2_H_2_^+^, *m/z* = 28; ^13^C_2_H^+^, *m/z* = 27; and ^13^C_2_^+^, *m/z* = 26) is different. It is still consistent with the ratio of the non-isotope-labeled standards C_2_H_4_ (Supplementary Fig. 11) and C_2_H_6_ (Supplementary Fig. 12), respectively, which comply with the mass shift effect (*M*+2) caused by two labeled carbon atoms. In addition, another set of peaks that recorded from *m/z* = 13 to 16 exist both in the MS of ^13^C_2_H_4_ (^13^CH_2_^+^, *m/z* = 15; ^13^CH^+^, *m/z* = 14; ^13^C^+^, *m/z* = 13) and ^13^C_2_H_6_ (^13^CH_3_^+^, *m/z* = 16; ^13^CH_2_^+^, *m/z* = 15; ^13^CH^+^, *m/z* = 14; ^13^C^+^, *m/z* = 13), which can be attributed to fragments caused by broken carbon–carbon bonds.

When the potential products of CO_2_ photoreduction are liquids such as alcohols or acids, we used the headspace injection method instead of airtight needles (see “Methods” for experimental details). The temperature of the HP-PLOT/Q column was raised to 120 °C to separate alcohols. The liquids suspected to be methanol or CH_3_OH (RT at 6.14 min) and ethanol or CH_3_CH_2_OH (RT at 12.23 min) eluted after the air components (RT at 2.96 min), CO_2_ (RT at 3.18 min), and H_2_O (RT at 3.98 min) in TIC (Fig. 4c). The material at RT = 6.14 min was identified as ^13^CH_3_OH based on two sets of peaks: one set at *m/z* = 29 to 33 and another set at *m/z* = 13 to 16 attributed to the molecular ions of ^13^CH_3_OH^+^ (*m/z* = 33) and the fragment ions of ^13^CH_3_O^+^ (*m/z* = 32), ^13^CH_2_O^+^ (*m/z* = 31), ^13^CHO^+^ (*m/z* = 30), ^13^CHO^+^ (*m/z* = 29), ^13^CH_3_^+^ (*m/z* = 16), ^13^CH_2_^+^ (*m/z* = 15), ^13^CH^+^ (*m/z* = 14), and ^13^C^+^ (*m/z* = 13), respectively. It is important to note that the largest peak in the MS was ^13^CH_3_O^+^ (*m/z* = 32) due to the easier dissociation of CH_3_OH. It also exhibits a mass shift effect (*M*+1) compared to the non-isotope-labeled CH_3_OH (Supplementary Fig. 13). Despite the fact that ^13^CH_3_^13^CH_2_OH also has its largest fragment ion peak at *m/z* = 32, the source of this peak is derived from the cleavage of the carbon–carbon bonds, generating hydrogenated ^13^CH_2_OH^+^ instead of ^13^CH_3_O^+^ as was the case for ^13^CH_3_OH. Moreover, there is no distinct peak at *m/z* = 33 in the MS of ^13^CH_3_^13^CH_2_OH that matches ^13^CH_3_OH. The MS of the ethanol fraction ^13^CH_3_^13^CH_2_OH also has a set of peaks at *m/z* from 43 to 48, matching the molecular ions of ^13^C_2_H_5_OH^+^ (*m/z* = 48) and fragment ions of ^13^C_2_H_4_O^+^ (*m/z* = 47), ^13^C_2_H_3_O^+^ (*m/z* = 46), ^13^C_2_H_2_O^+^ (*m/z* = 45), ^13^C_2_HO^+^ (*m/z* = 44), and ^13^C_2_O^+^ (*m/z* = 43). It exhibited a mass shift effect of (*M*+2) or (*M*+1) versus the non-isotope-labeled C_2_H_5_OH (Supplementary Fig. 14) and the single-isotope-labeled CH_3_^13^CH_2_OH (Supplementary Fig. 15), respectively, due to the two isotope-labeled carbon atoms in one molecule of ^13^CH_3_^13^CH_2_OH (Supplementary Fig. 16). All of these points mentioned above are sufficient enough to match the qualitative requirements for ^13^CH_3_OH and ^13^CH_3_^13^CH_2_OH. Likewise, the potential products of carboxylic acids (HCOOH and CH_3_COOH) could be separated by choosing a column (HP-FFAP) with stronger polarity (see “Methods” for experimental details). As shown in Fig. 4d, separation depends on the different molecular polarities between CH_3_COOH, HCOOH, and the reactants of H_2_O and CO_2_. CH_3_COOH has a relatively short retention time (RT at 8.07 min) than HCOOH (RT at 10.17 min), which elutes from the column after the air components (RT at 2.62 min) and H_2_O (RT at 3.15 min). By analyzing the corresponding MS, the non-isotope-labeled CH_3_COOH (Supplementary Fig. 17) possesses a molecular ion peak at *m/z* = 60 and two prominent fragment ion peaks at *m/z* = 43 and *m/z* = 45, while the corresponding molecular ion and fragment ions peak obtained from isotope-labeled ^13^CH_3_^13^COOH shift to *m/z* = 62, *m/z* = 45, and *m/z* = 46, respectively. It reveals a difference in relative proportion compared to non-isotope-labeled CH_3_COOH, even considering the isotope-induced mass shift effect (*M*+2). After checking the MS of CH_3_^13^COOH (Supplementary Fig. 18), a molecular ion peak at *m/z* = 61 and two fragment ion peaks at *m/z* = 44 and *m/z* = 46 were obtained. The two fragment ions of CH_3_^13^COOH (*m/z* = 44 and *m/z* = 46), as well as the corresponding fragment ions of ^13^CH_3_^13^COOH (*m/z* = 45 and *m/z* = 46) and CH_3_COOH (*m/z* = 43 and *m/z* = 45), are generated by carbon–carbon bond cleavage (COOH^+^) and dehydroxylation (CH_3_CO^+^), respectively (Supplementary Fig. 19). In addition, the isotope-labeled H^13^COOH possesses H^13^COOH^+^ (*m/z* = 47) molecular ions and fragment ions including ^13^COOH^+^ (*m/z* = 46), ^13^COO^+^ (*m/z* = 45), H^13^CO^+^ (*m/z* = 30), and ^13^CO^+^ (*m/z* = 29), which exhibit a mass shift effect (*M*+1) compared to the non-isotope-labeled HCOOH (Supplementary Fig. 20).

**Fig. 5:**
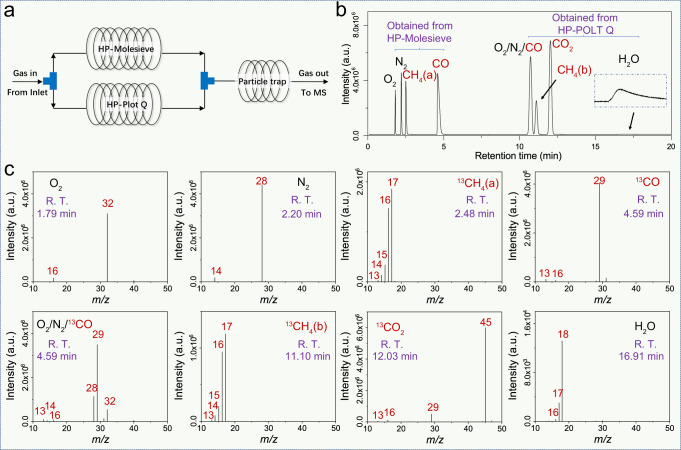
A developed strategy of parallel connection system. **a** Scheme illustrating a parallel connection system using HP-Molesieve and HP-PLOT/Q columns. The TIC (**b**) and the corresponding MS spectra (**c**) of a sample containing O_2_, N_2_, CO, CH_4_, CO_2_, and water vapor. Source data are provided as a Source Data file.

The four methods mentioned above can identify reactants and products in CO_2_ photoreduction reactions. However, this process requires multiple setups, and there is no standard strategy to separate and analyze all these products, reactants, and air components simultaneously. Even for the most reported photocatalytic CO_2_ conversion process of CO_2_ to CO, a mixture of O_2_, N_2_, CO, CH_4_, CO_2_, and water vapor cannot be completely separated with one column due to the irreversible adsorption of CO_2_ in the HP-Molesieve column and poor separation of CO/N_2_ in the HP-PLOT/Q column. To solve this problem, we designed a parallel connection system (Fig. 5a, see “Methods” for experimental details) containing both HP-Molesieve and HP-PLOT/Q columns. The diameters of the parallel GC columns were optimized to enable the components from different columns to queue into the MS detector separately. As shown in Fig. 5b, O_2_ (RT at 1.79 min), N_2_ (RT at 2.20 min), CH_4_ (RT at 2.48 min), and CO (RT at 4.59 min) elute from HP-Molesieve one by one and generate the corresponding MS spectra (Fig. 5c). It matches the abovementioned standard MS in Fig. 4a, respectively. CO_2_ and H_2_O could not be resolved by the HP-Molesieve channel but could be measured in the parallel HP-PLOT/Q channel. They eluted in the HP-PLOT/Q channel as an N_2_/O_2_/CO mixture (RT at 10.68 min), CH_4_ (RT at 11.10 min), CO_2_ (RT at 12.03 min), and H_2_O (RT at 16.91 min), which matches the standard MS in Fig. 4b. Based on this parallel strategy, the simulated mixture from reagents to products to air components in the potential photocatalytic CO_2_ reduction process could be analyzed in one GC-MS experiment.

### Testing the isotope-tracing protocol using samples generated by well-studied CO_2_ photoreduction reactions

**Fig. 6:**
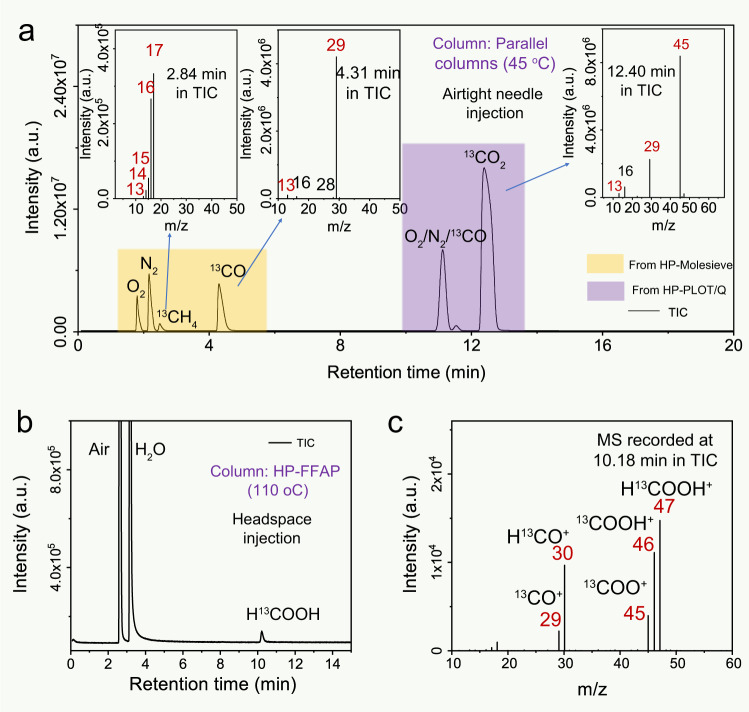
Isotope-tracing experiments in reported homogeneous and heterogeneous photoreduction systems. GC-MS analytical data for ^13^C isotope-tracing experiments in CO_2_ photoreduction using the following catalysts: Fe^III^ porphyrin complexes in homogeneous phase (**a**) and Cd-doped ZnS in heterogeneous phase (**b**, **c**). Each panel shows the TICs and MS spectra of products generated by the catalysts in a CO_2_ photoreduction reaction. Source data are provided as a Source Data file.

After optimizing our isotope-tracing methods using multiple standard samples, we tried to verify its reliability using some previously reported photoreduction systems. The homogeneous molecular catalyst Fe^III^ porphyrin complex was used to evaluate CO_2_ photoreduction (see “Methods” for experimental details) in a closed gas circulation system (Supplementary Fig. 21)^59^. As shown in Fig. 6a, the obtained TIC of this system is similar to the standard spectra (Fig. 4a**)** because CH_4_ and CO are products of this reaction. In MS, the molecular ion peaks, as well as the fragment ion peaks, were assigned to the isotopically labeled ^13^CH_4_ and ^13^CO, respectively, conclusively demonstrating that the products of CH_4_ and CO originated from the photoreduction of labeled ^13^CO_2_ (Supplementary Fig. 22). Bipyridine complexes of ruthenium (Ru(bpy)_3_Cl_2_) is also a common homogeneous catalyst for CO_2_ photoreduction. By comparison with the sample without reaction (Supplementary Fig. 23), we could confirm that the ^13^CO product comes from the reduction of CO_2_ but not from the interfering factor of reactants (fragment ions from ^13^CO_2_) (see “Methods” for experimental details). We also examined CO_2_ photoreduction using a heterogeneous reaction system with Cd-doped ZnS as the photocatalyst. Cd-doped ZnS can reduce CO_2_ into HCOOH under light irradiation and was used to validate our GC-MS isotope-tracing method for liquid-phase products (see “Methods” for experimental details)^60^. Fig. 6 shows the TIC (Fig. 6c) and MS spectrum (Fig. 6d) collected using the products. The peak at RT = 10 min in the TIC corresponded to three peaks in the MS that match our standard MS for H^13^COOH (Fig. 4b). These results demonstrate that our method is also capable of tracing liquid products generated by CO_2_ reduction.

**Fig. 7:**
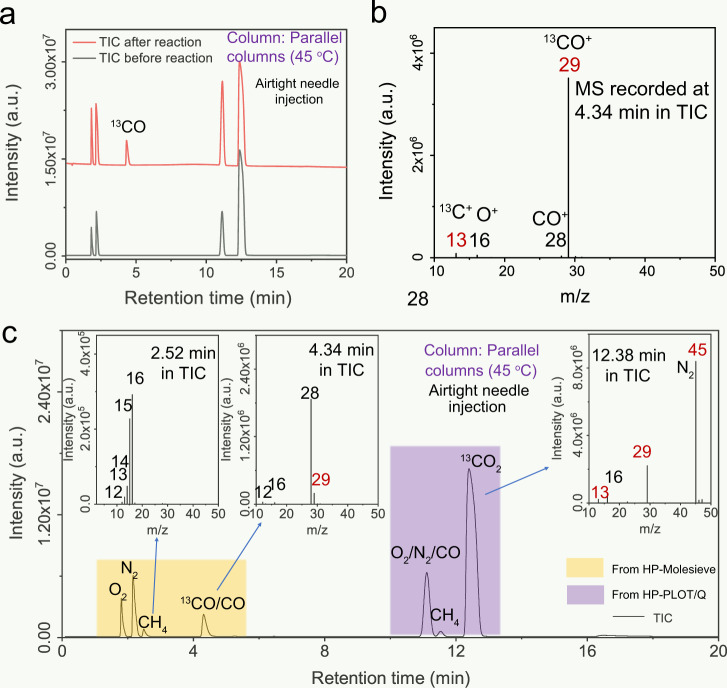
Isotope-tracing experiments in reported liquid and gas–solid phase photoreduction systems. GC-MS analytical data for ^13^C isotope-tracing experiments in CO_2_ photoreduction using the following catalysts: conjugated polymers in liquid (**a**, **b**) and gas–solid phase (**c**). Each panel shows the TICs and MS spectra of products generated by the catalysts in a CO_2_ photoreduction reaction. Source data are provided as a Source Data file.

In addition, isotope tracing in all-organic reaction systems is particularly difficult to assess in CO_2_ photoreduction experiments. We employed our recently reported conjugated polymers (CPs) as a representative photocatalyst to experiment with both gas–solid phases (Supplementary Fig. 24) (see “Methods” for experimental details) and liquid phase (Supplementary Fig. 25) (see “Methods” for experimental details) reactions^34^. Only an increase in CO could be observed during the liquid phase reaction by comparing the TIC before and after photoreduction (Fig. 7a), and the MS collected at RT = 4.59 min matches the standard MS of ^13^CO (Fig. 7b), implying that CO originates from CO_2_ reduction (Supplementary Fig. 26). In contrast, CPs in the gas phase reaction possesses much higher activities and produces additional CH_4_ compared to the liquid phase reaction. In the corresponding MS (inset of Fig. 6c), CO and CH_4_ are primarily generated by photothermal effect-induced decomposition on solid CP instead of CO_2_ photoreduction (Supplementary Fig. 27). This result leaves little doubt that the self-decomposition of catalyst can seriously affect both the qualitative and quantitative analysis of the product when evaluating the CO_2_ photoreduction in the gas-solid phase of CPs. It also illustrates the importance of accurate isotope-tracing methods to prove the efficiency of CO_2_ photoreduction in different materials systems. Moreover, it should be emphasized that the isotope experiments and accurate GC-MS tests are not only necessary for the reaction systems using sacrificial agents but also important for the reaction systems without sacrificial agents.

## Discussion

In summary, we initially demonstrated the difficulty of accurately ascribing products of CO_2_ photoreduction in isotope-tracing experiments. Existing methods are known to be rather crude due to the similarity of reactants, products, and the catalyst itself, which negatively affects efforts toward reliable CO_2_ photoreduction processes. Thus we sought to develop a rigorous strategy to eliminate the false-positive results by providing solid evidence of CO_2_ photoreduction. We disclosed many often neglected false-positive results in isotope-tracing studies using standard isotopically labeled molecules and the basic principles of GC-MS analysis. Through extensive testing, we systematically presented isotope-tracer experiments methods of standard spectra for various potential products of CO, alkanes, alcohols, carboxylic acids, and alkenes. Then we devised a method to simultaneously analyze these potential products using a parallel connection system using two GC columns. The accuracy of this parallel setup was verified with the standard isotopes described earlier; then, the setup was used to analyze four previously reported systems for CO_2_ photoreduction. The purpose of this research is to highlight appropriate scientific methods in CO_2_ photoreduction, help researchers avoid the pitfalls and misunderstandings in isotope-tracing experiments, and propose some standard procedures. More precise procedures will allow researchers to provide better feedback in their efforts to design photocatalysts with high selectivities and conversion efficiencies for CO_2_ photoreduction. We also provide examples and reference measurements for the ^13^C isotopes of numerous known reactants and products to help researchers firmly attribute them to CO_2_ reduction reactions.

## Methods

### Using HP-5ms as a column for isotope-labeled samples analysis

The 0.5 ml gas samples were collected and injected by gas-tight syringes (the VICI Pressure-Lok Precision Analytical Syringe A-2 Series (050033), 1 ml) and then analyzed by gas chromatography-mass spectrometry (8890-5977B GC-MS instrument, Agilent Technologies, USA) equipped with most used commercial capillary columns (HP-5ms, 5%-Phenyl-methylpolysiloxane, 19091S-433UI-KEY, 30 m × 0.25 mm × 25 μm, Agilent Technologies, USA) in GC-MS. Helium was used as carrier gas. The column was maintained at 150 °C for 25 min, and the flow of the carrier was 0.8 ml l^−^^1^. The temperatures of the injector, EI source, and GCITF were set to be 200, 200, and 250 °C, respectively. The selected mass-to-charge ratios of ions were 17, 29, and 45 in SIM mode. Developing a suitable programmed temperature rise process can further shorten the detection time.

### Using SVUV-PIMS for isotope-labeled samples analysis

The synchrotron VUV photoionization mass spectroscopy (SVUV-PIMS) experiments were performed on the combustion station of the National Synchrotron Radiation Laboratory (Hefei, China). The test modes are the photoionization energy scan under the determined mass-to-charge ratio and the mass-to-charge ratio scan under the determined photoionization energy, respectively. Synchrotron radiation from the undulator beamline was monochromatized with 200 lines/mm laminar grating (Horiba Jobin Yvon, France), which covered the photon energy from 7.5 to 22 eV with an energy resolving power of 3000 (E/∆E at 10 eV). The average photon flux could reach the magnitude of 1013 photons/s after suppressing the higher-order harmonic radiation by a gas filter filled with noble gas^61,62^.

### Using different columns for isotope-labeled samples analysis

The 0.5 ml gas samples were collected and injected by gas-tight syringes (the VICI Pressure-Lok Precision Analytical Syringe A-2 Series (050033), 1 ml), and the 2 ml liquid samples were collected and placed in a headspace sampler; then the samples were analyzed by gas chromatography-mass spectrometry (8890-5977B GC-MS instrument, Agilent Technologies, USA) equipped with commercial capillary columns. The column was maintained at a certain temperature for 15 min, and the flow of the carrier was 0.8 ml l^−^^1^. The temperatures of the injector, EI source, and GCITF were set to be 200, 200, and 250 °C, respectively. The mass-to-charge ratio of the mass scanning mode was set from 2 to 70. The GC-MS was operated the post-run after each injection (the temperature of the column oven increased to 300 °C with a rate of 30 °C and then maintained at 300 °C for 10 min). Developing a suitable programmed temperature rise process can further shorten the detection time.

The information of the columns are listed below:

(HP-Molesieve, 5A molesieve, 19091S-MS8, 30 m × 0.32 mm × 25 μm, Agilent Technologies, USA; HP-PLOT/Q, Bonded polystyrene-divinylbenzene, 19091P-QO4, 30 m × 0.32 mm × 20 μm, Agilent Technologies, USA; HP-FFAP, Modified polyethylene glycol, 19091F-413, 30 m × 0.32 mm × 20 μm, Agilent Technologies, USA).

### Using a designed parallel connection system as a column for isotope-labeled samples analysis

The 0.5 ml gas samples were collected and injected by gas-tight syringes (the VICI Pressure-Lok Precision Analytical Syringe A-2 Series (050033), 1 ml) and then analyzed by gas chromatography-mass spectrometry (8890-5977B GC-MS instrument, Agilent Technologies, USA) equipped with designed parallel connection system (HP-Molesieve 15 m × 0.53 mm × 20 μm, HP-PLOT/Q 15 m × 0.32 mm × 20 μm, and CP4016 10 m × 0.32 mm, Agilent Technologies, USA) in GC-MS. Helium was used as carrier gas. The column was maintained at 45 °C for 20 min, and the flow of the carrier was 0.8 ml l^−^^1^. The temperatures of the injector, EI source, and GCITF were set to be 200, 200, and 250 °C, respectively. The mass-to-charge ratio of the mass scanning mode was set from 2 to 70. The GC-MS was operated the post-run after each injection (the temperature of the column oven increased to 300 °C with a rate of 30 °C and then maintained at 300 °C for 10 min). Developing a suitable programmed temperature rise process can further shorten the detection time.

## Supplementary information

Supplementary Information

Peer Review File

## Data Availability

The data that support the plots within this paper and other findings of this study are available from the corresponding author upon reasonable request. Source data are provided with this paper.

## Source data

Source Data
