# Supplementary material for: Designing reliable and accurate isotope-tracer experiments for CO2 photoreduction
Source: Nat Commun. 2023 May 3;14:2534. doi: 10.1038/s41467-023-38052-0 (PMC10156805; doi:10.1038/s41467-023-38052-0)
Supplement: Supplementary file 1 — Supplementary Information [file 41467_2023_38052_MOESM1_ESM.pdf]

Supplementary Information

Realizing Reliable and Accurate Isotope-Tracer Experiments in CO_2 Photoreduction

by Wang et al.

Supplementary Methods

Chemicals

All reagents were purchased from Sigma-Aldrich or Sinopharm Chemical Reagent Co., Ltd without further purification.

All isotope labeled reagents were purchased from Sigma-Aldrich or Cambridge Isotope Laboratories, Inc. without further purification.

The high purity helium (Chemical Purity 99.999%) with further purification by helium purifier (HP2, Valco Instruments Co. Inc) were used as the carrier gas for GC-MS.

^{13}CO (CLM-189-PK, Chemical Purity 99.5%, Isotope purity, 99%, Cambridge Isotope Laboratories, Inc.); **$^{13}CO_2$** (486418, Chemical Purity 99.9%, Isotope purity 99%, Sigma-Aldrich); **$^{13}CH_4$** (CLM-429-PK, Chemical Purity 99%, Isotope purity, 99%, Cambridge Isotope Laboratories, Inc.); **$^{13}C_2H_6$** (489220, Chemical Purity 99.5%, Isotope purity 99%, Sigma-Aldrich); **$^{13}C_2H_4$** (CLM-415-PK, Chemical Purity 98%, Isotope purity 99%, Sigma-Aldrich); **$^{13}CH_3OH$** (277177, Chemical Purity 99%, Isotope purity 99%, Sigma-Aldrich); **$^{13}CH_3{}^{13}CH_2OH$** (427039, Chemical Purity 99%, Isotope purity 99%, Sigma-Aldrich); **$CH_3{}^{13}CH_2OH$** (324523, Chemical Purity 99%, Isotope purity 99%, Sigma-Aldrich); **$H^{13}COOH$** (CLM-1284-PK, Chemical Purity 99%, Isotope purity 99%, Cambridge Isotope Laboratories, Inc.); **$^{13}CH_3{}^{13}COOH$** (279307, Chemical Purity 99%, Isotope purity 99%, Sigma-Aldrich); **$CH_3{}^{13}COOH$** (279285, Chemical Purity 99%, Isotope purity 99%, Sigma-Aldrich).

Synthesis of Fe^{III} porphyrin complex

A solution of commercial 5,10,15,20-tetra(4′-N,N,N-trimethylanilinium) porphyrin tetrachloride (51 mg), anhydrous iron (II) bromide (201 mg), and 2,6-lutidine (60 μL) was

degassed by argon for 15 min; the mixture was stirred at reflux under inert atmosphere for 7 d. After methanol was removed, the resulting solid was sonicated in THF and filtered. The brown solid on the glass frit was washed with dichloromethane and dissolved in methanol; after the mixture was concentrated under reduced pressure, a small amount of concentrated HCl was added with a large quantity of THF to give Fe^{III} porphyrin complex as a dark red powder.

Synthesis of CPs

The CPs were synthesized according to Suzuki-Miyaura cross-coupling polycondensation. In details, a dry 250 mL round-bottom flask was charged with 1,4-phenylenediboronic acid (248 mg, 1.5 mmol), 1,3,6,8-Tetrabromopyrene (388 mg, 0.75 mmol), $Pd(PPh_3)_4$ (10 mg), K_2CO_3 (2.0 g), and mixed solvent of dimethyl formamide (60 mL)/water (8 mL). The mixture was degassed by bubbling with Ar for 30 min and then the resulting mixture was stirred at 150 °C for 24 h under Ar condition. After that, the precipitate was collected by filtration, and the solid was washed with methanol and CH_2Cl_2 in the Soxhlet for 48 h. The final product was dried at 60 °C overnight.

Synthesis of Cd-doped ZnS

The colloidal ZnS (0.1g) can be quite efficiently obtained by directly pouring the freshly prepared Na_2S solution (1.026 mmol in 20 ml) into ZnSO4 solution (1.026 mmol in 20 ml) under vigorous stirring followed by centrifugation (10,000 rpm, 2 min). The Cd-doped ZnS can be obtained by adding a certain amount of Cadmium sulfate solution for ion exchange. Extending stirring time and further hydrothermal or heat treatment are not needed. Then, the received precipitate was redispersed in water for activity test in the reaction cell without drying

procedure.

Methods

Isotope labeling measurement for Fe^{III} porphyrin complex, Ru complex and CPs

For the system of *Fe^{III} porphyrin complex,* the *Fe^{III} porphyrin complex* was dissolved in the acetonitrile/water solutions containing triethylamine as sacrificial electron donor, and $Ir(ppy)_3$ were employed as sensitizer; For the system of *Ru complex,* the *Ru complex* was dissolved in the acetonitrile/water solutions containing triethylamine as sacrificial electron donor; For the gas-solid system of *CPs, CPs* powders were uniformly dispersed onto a porous quartzose film which was fixed on the stage inside the reaction cell with addition of 3 mL of distilled water as electron donor; For the liquid system of *CPs, CPs* powders dispersed in the acetonitrile/water solutions containing triethylamine as sacrificial electron donor.

The isotope labeling measurement was carried out by using $^{13}CO_2$ gas (486418, Chemical Purity 99.9%, Isotope purity 99%, Sigma-Aldrich) instead of pure $^{12}CO_2$ gas (Chemical purity, 99.999%, Newradar Gas Co., Ltd.) as the carbon source with the same reaction and the gas products were analyzed by gas chromatography-mass spectrometry (8890-5977B GC-MS instrument, Agilent Technologies, USA) equipped with designed parallel connection system. Helium was used as carrier gas. The column was maintained at 45 °C for 20 min, and the flow of the carrier was 0.8 ml L^{-1}. The temperatures of the injector, EI source, and the GCITF were set to be 200, 200, and 250 °C, respectively. Developing a suitable programmed temperature rise process can further shorten the detection time.

Isotope labeling measurement for Cd-doped ZnS

The reaction solution contains 0.1 g colloidal Cd-ZnS and 0.1 mol L^{-1} K_2SO_3. The isotope

labeling measurement was carried out by using $^{13}CO_2$ gas (486418, Chemical Purity 99.9%, Isotope purity 99%, Sigma-Aldrich) instead of pure $^{12}CO_2$ gas (Chemical purity, 99.999%, Newradar Gas Co., Ltd.) as the carbon source and the liquid samples were collected and placed in headspace sampler, and then analyzed by gas chromatography-mass spectrometry (8890-5977B GC-MS instrument, Agilent Technologies, USA) equipped with HP-FFAP in GC-MS. The Helium was used as carrier gas. The column was maintained at 110 °C for 15 min, and the flow of the carrier was 0.8 ml L^{-1}. The temperatures of the injector, EI source, and the GCITF were set to be 200, 200, and 250 °C, respectively. The mass-to-charge ratio of mass scanning mode were set from 2 to 70. Developing a suitable programmed temperature rise process can further shorten the detection time.

Supplementary Figures

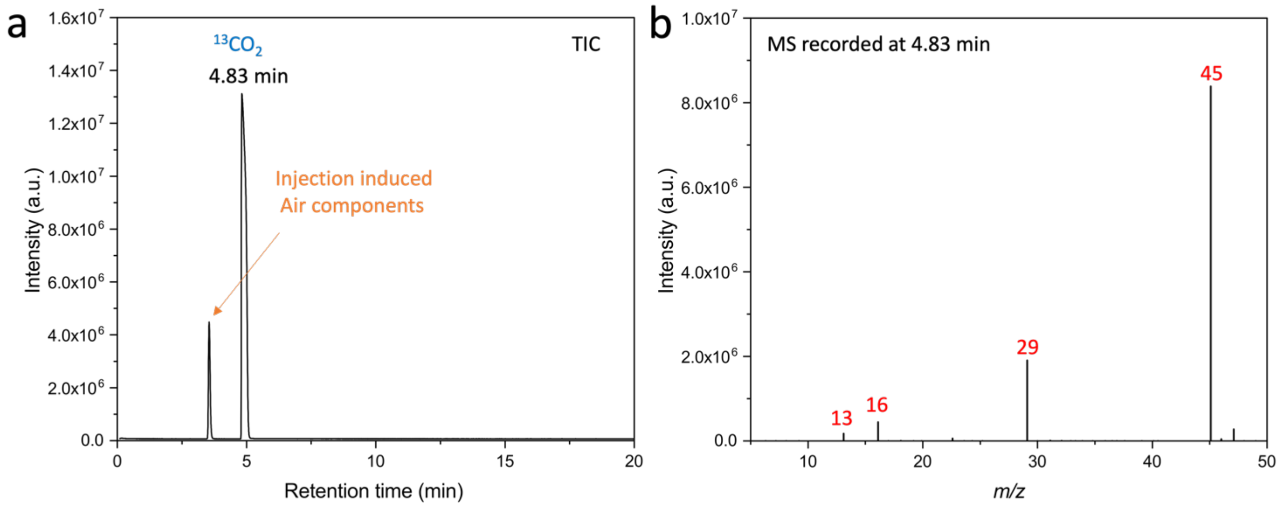

Figure S1. The TIC spectra of $^{13}CO_2$ (a) and the corresponding MS spectra (b) recorded at 4.83 min in TIC.

The molecular ion of $^{13}CO_2$ ($^{13}CO_2^+$, *m/z* = 45) and the fragment ions of $^{13}CO_2$ ($^{13}CO^+$, *m/z* = 29; C^+, *m/z* = 16;$^{13}C^+$, *m/z* = 13) could be obtained from the MS spectra of $^{13}CO_2$.

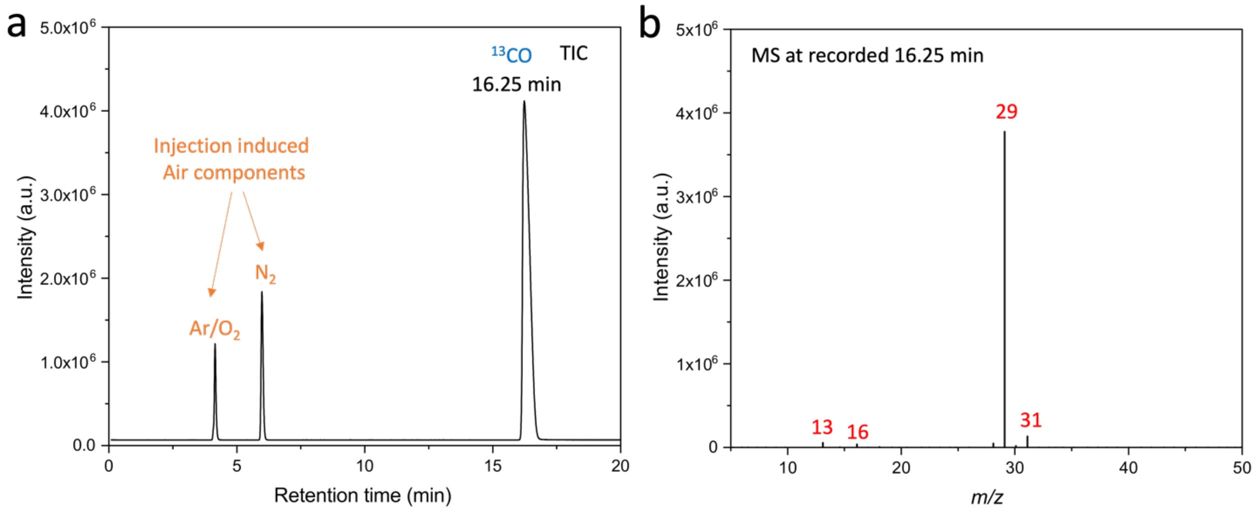

Figure S2. The TIC spectra of ^{13}CO (a) and the corresponding MS spectra (b) recorded at 16.25 min in TIC.

The molecular ion of ^{13}CO ($^{13}CO_2^+$, *m/z* = 29) and the fragment ions of ^{13}CO (C^+, *m/z* = 16; $^{13}C^+$, *m/z* = 13) could be obtained from the MS spectra of ^{13}CO.

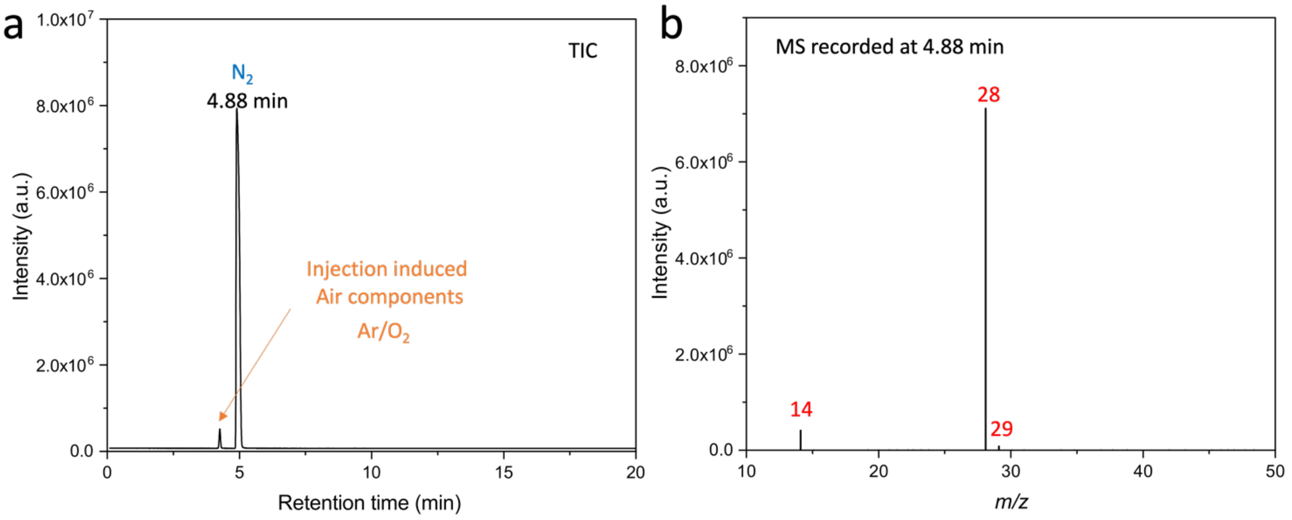

Figure S3. The TIC spectra of N_2 (a) and the corresponding MS spectra (b) recorded at 4.88 min in TIC.

The molecular ion of abundant N_2 (N_2^+, m/z = 28) and $^{14}N^{15}N$ ($^{14}N^{15}N^+$, m/z = 29) and the fragment ions of N_2 (N^+, m/z = 14;) could be obtained from the MS spectra of N_2.

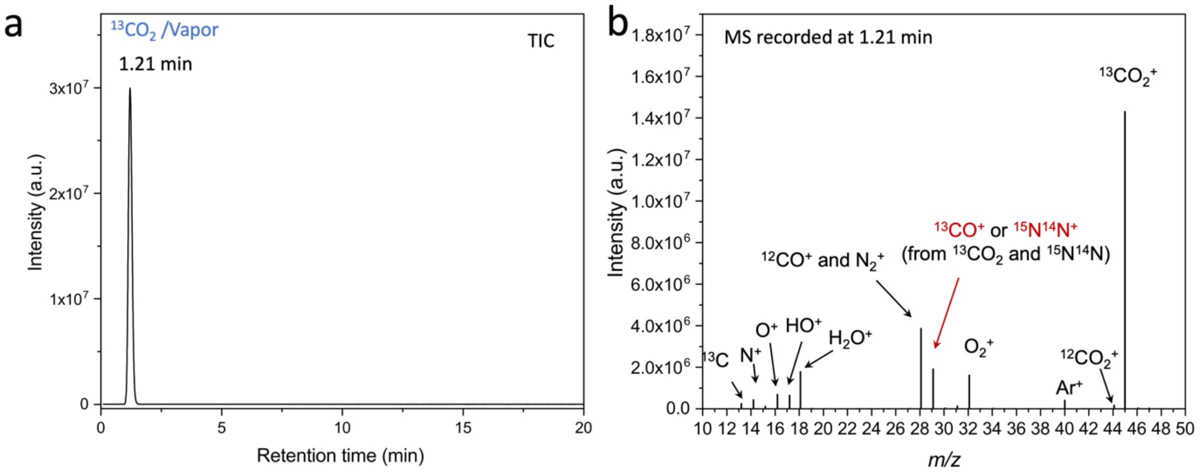

Figure S4. The TIC spectra of the $^{13}CO_2$ (a) and vapor mixture and the corresponding MS spectra (b) recorded at 1.21min in TIC with the deactivated fused silica tube (5m) as the connector.

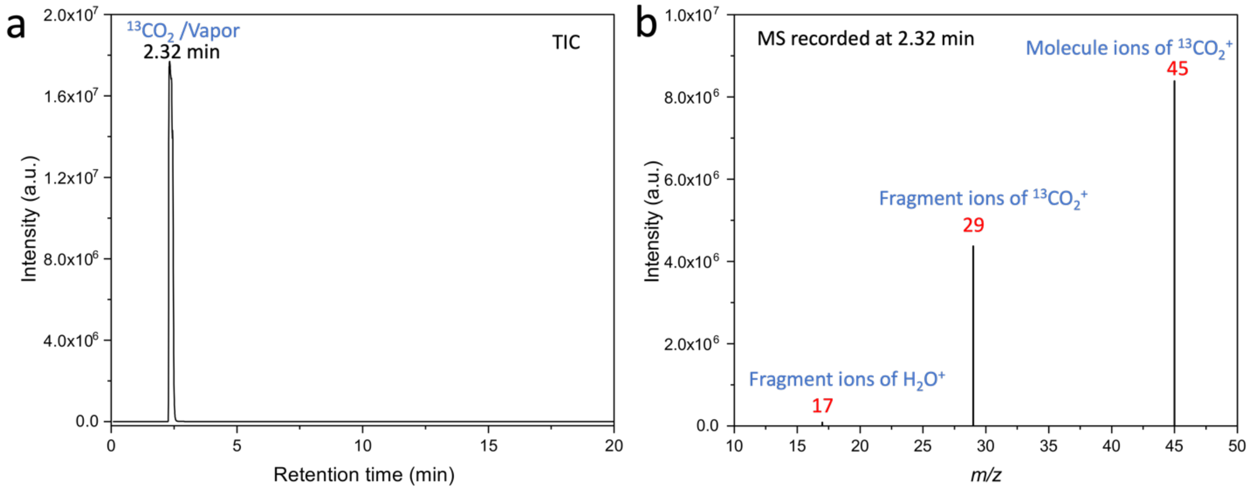

Figure S5. The TIC spectra of the $^{13}CO_2$ and vapor mixture (a) and the corresponding MS spectra (b) recorded at 2.32min in TIC under SIM mode.

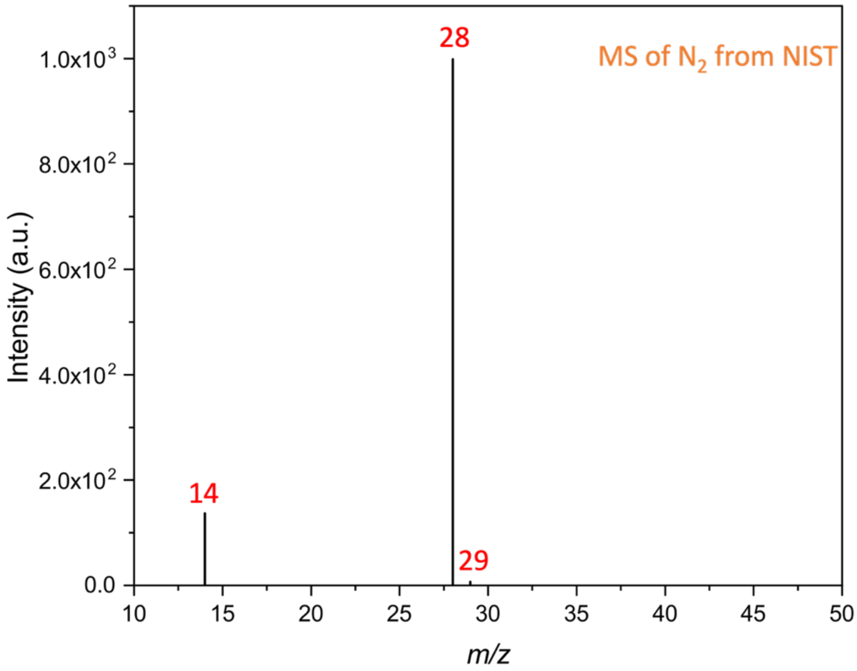

Figure S6. Standard MS spectra of N_2 from NIST library.

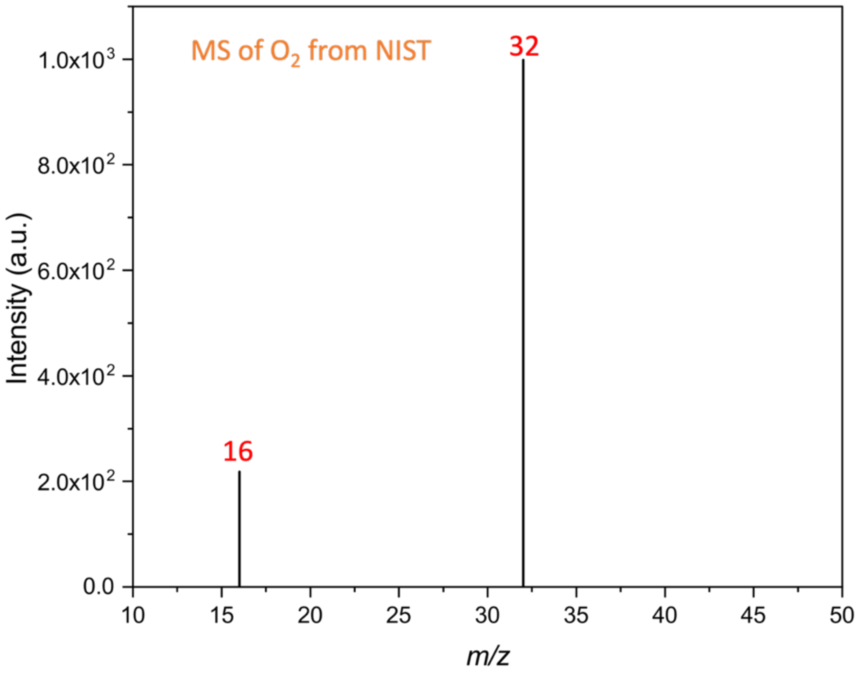

Figure S7. Standard MS spectra of O_2 from NIST library.

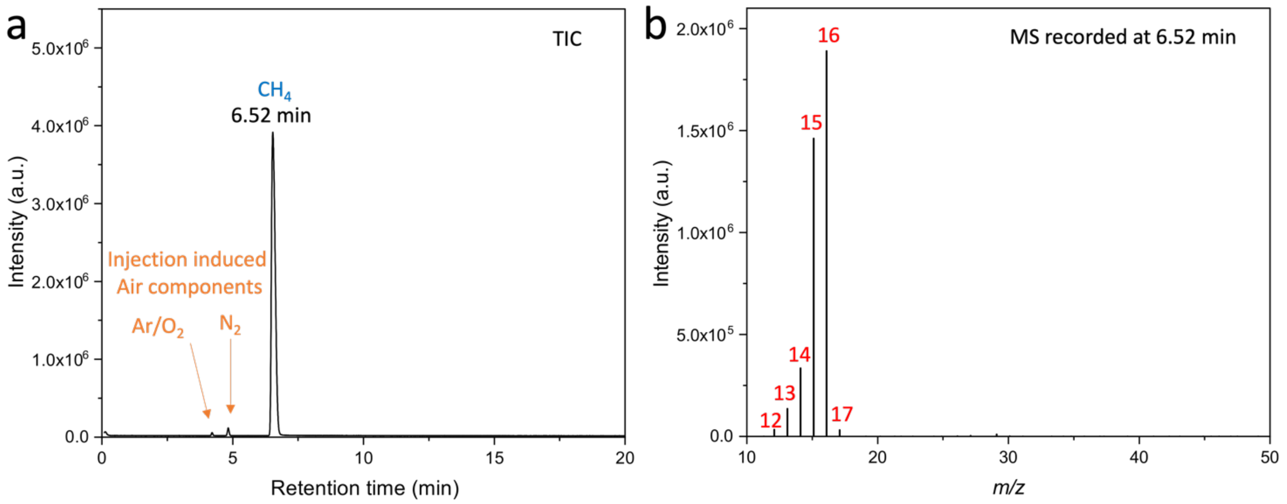

Figure S8. The TIC spectra of the CH_4 (a) and the corresponding MS spectra (b) recorded at 6.52min in TIC.

The non-isotope labeled also exhibits a peak at m/z=17, it could be attributed to the natural isotopic abundance of methane. As a result, there is no way to determine whether methane is a ^{13}C isotope labeled sample if the m/z=17 is detected in SIM method.

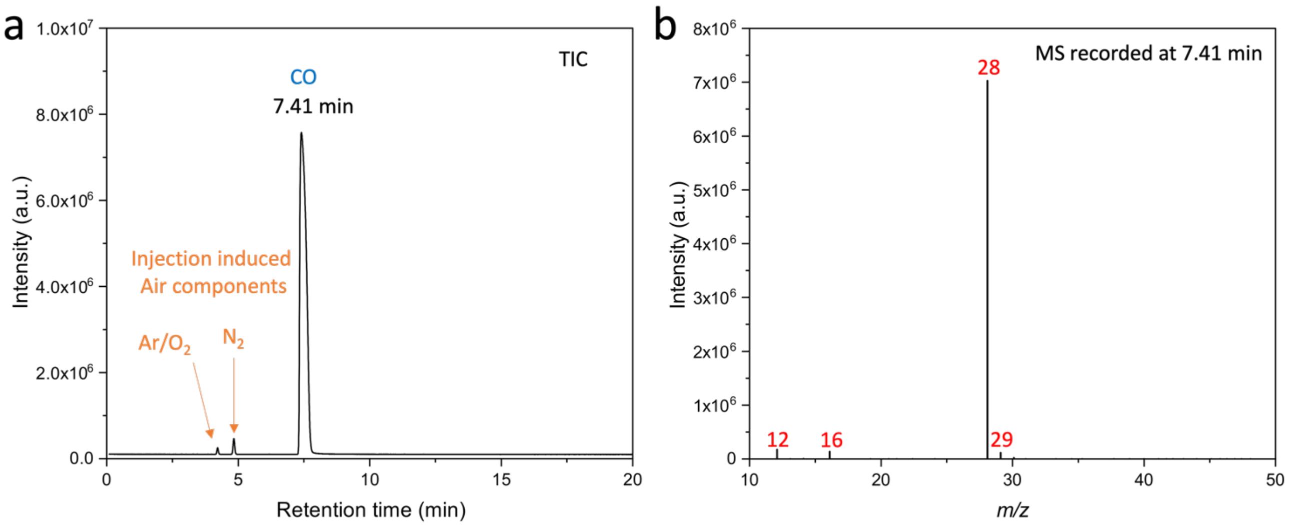

Figure S9. The TIC spectra of the CO (a) and the corresponding MS spectra (b) recorded at 7.41min in TIC.

The non-isotope labeled also exhibits a peak at m/z=29, it could be attributed to the natural isotopic abundance of carbon monoxide. As a result, there is no way to determine whether carbon monoxide is ^{13}C isotope labeled sample if the m/z=29 is detected in SIM method.

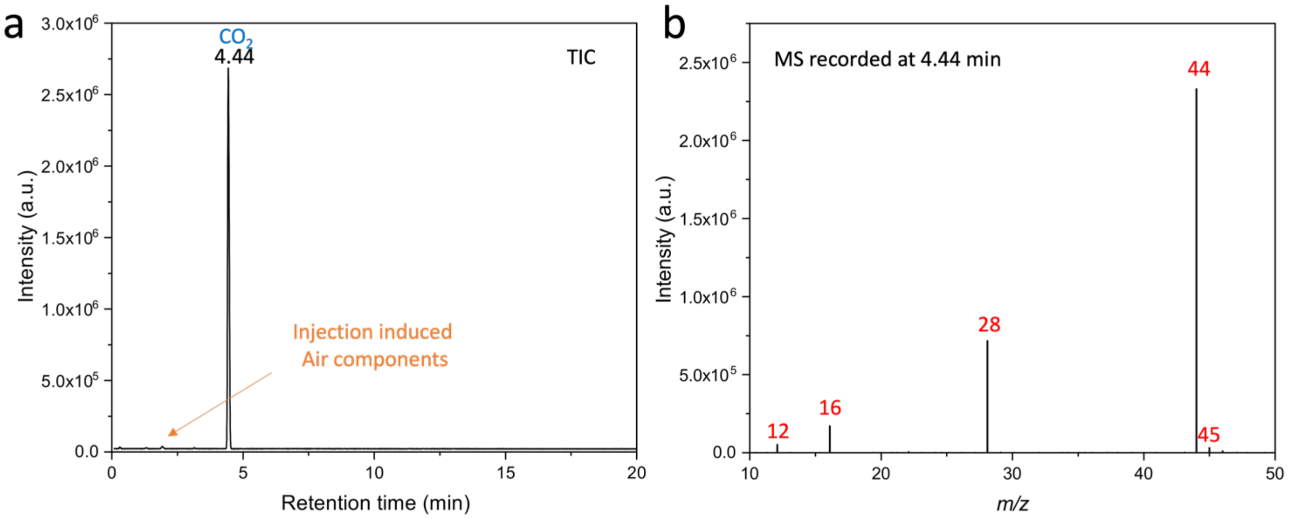

Figure S10. The TIC spectra of the CO_2 (a) and the corresponding MS spectra (b) recorded at 4.44min in TIC.

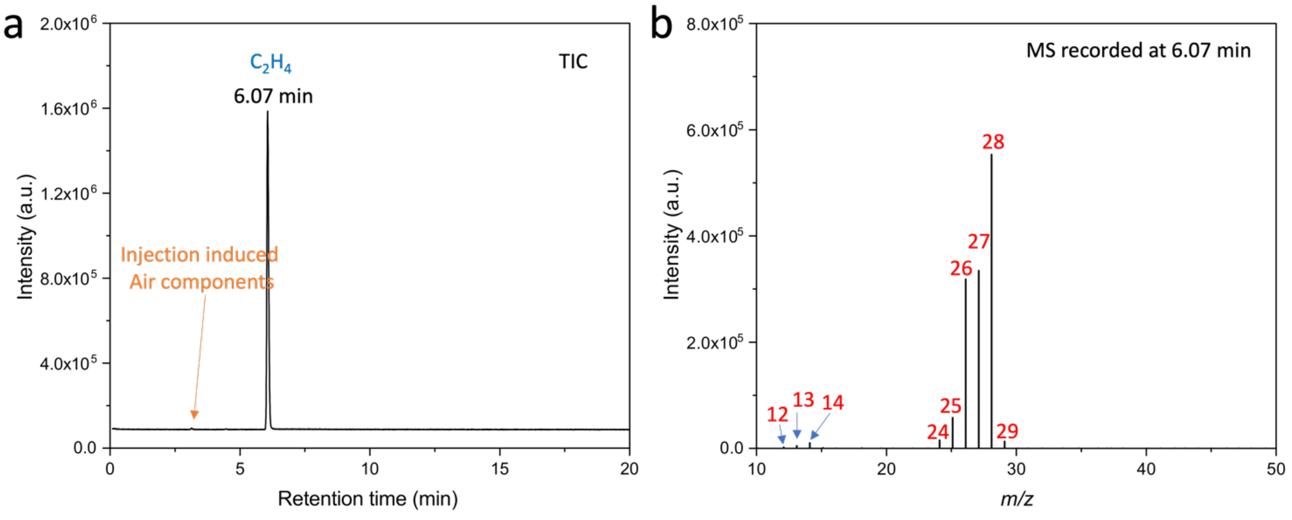

Figure S11. The TIC spectra of the C_2H_4 (a) and the corresponding MS spectra (b) recorded at 6.07 min in TIC.

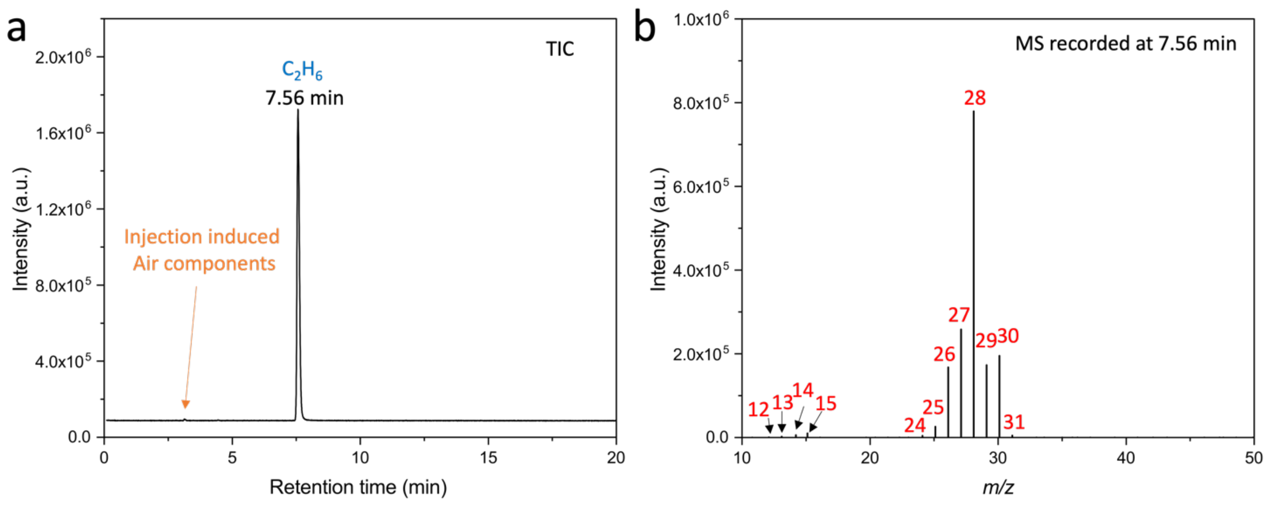

Figure S12. The TIC spectra of the C_2H_6 (a) and the corresponding MS spectra (b) recorded at 7.56 min in TIC.

Although the molecular ion peak of ethane is located at m/z=30, the fragment ion peak of C_2H_6 ($C_2H_4^+$, m/z=28) is much higher than molecular ion peak due to the easier C-H bond breaking process.

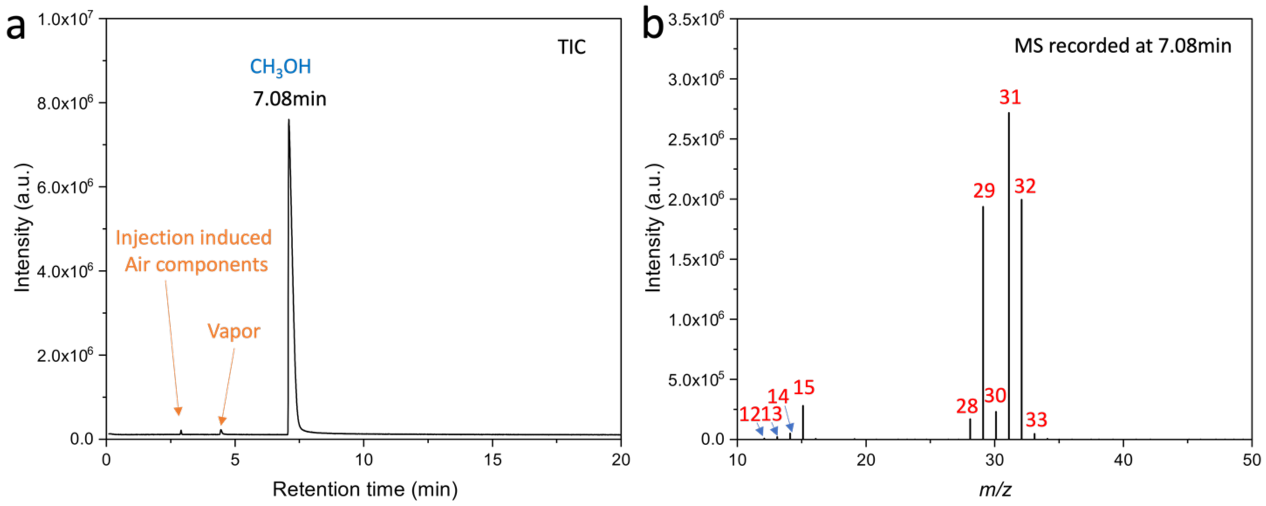

Figure S13. The TIC spectra of the CH_3OH (a) and the corresponding MS spectra (b) recorded at 7.08 min in TIC.

Although the molecular ion peak of ethane is located at m/z=32, the fragment ion peak of CH_3OH (CH_3O^+, m/z=31) is higher than molecular ion peak due to the hydrogen of the hydroxyl group is easily removed.

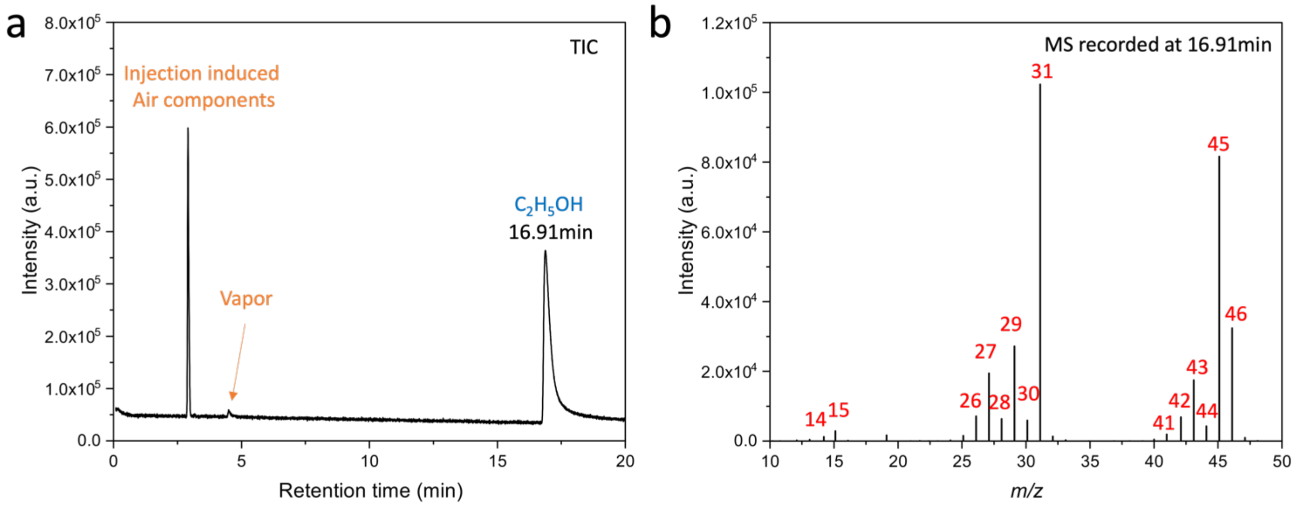

Figure S14. The TIC spectra of the C_2H_5OH (a) and the corresponding MS spectra (b) recorded at 16.91 min in TIC.

The highest peak in MS spectra of C_2H_5OH is the fragment ion of CH_2OH^+ (m/z=31) since the demethylation occurs easily during the ionization of ethanol.

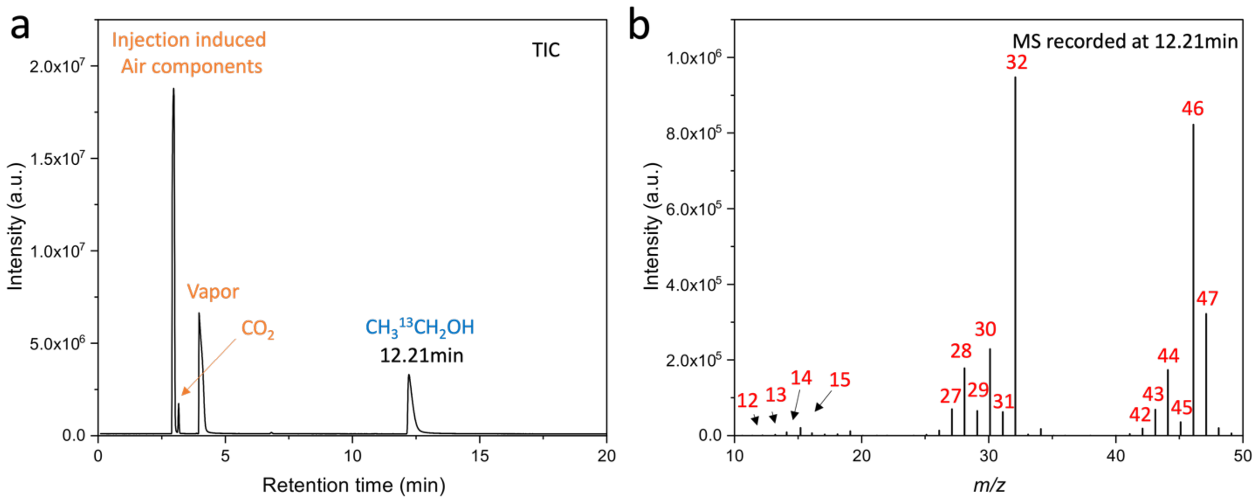

Figure S15. The TIC spectra of the $CH_3{}^{13}CH_2OH$ (a) and the corresponding MS spectra (b) recorded at 12.21 min in TIC.

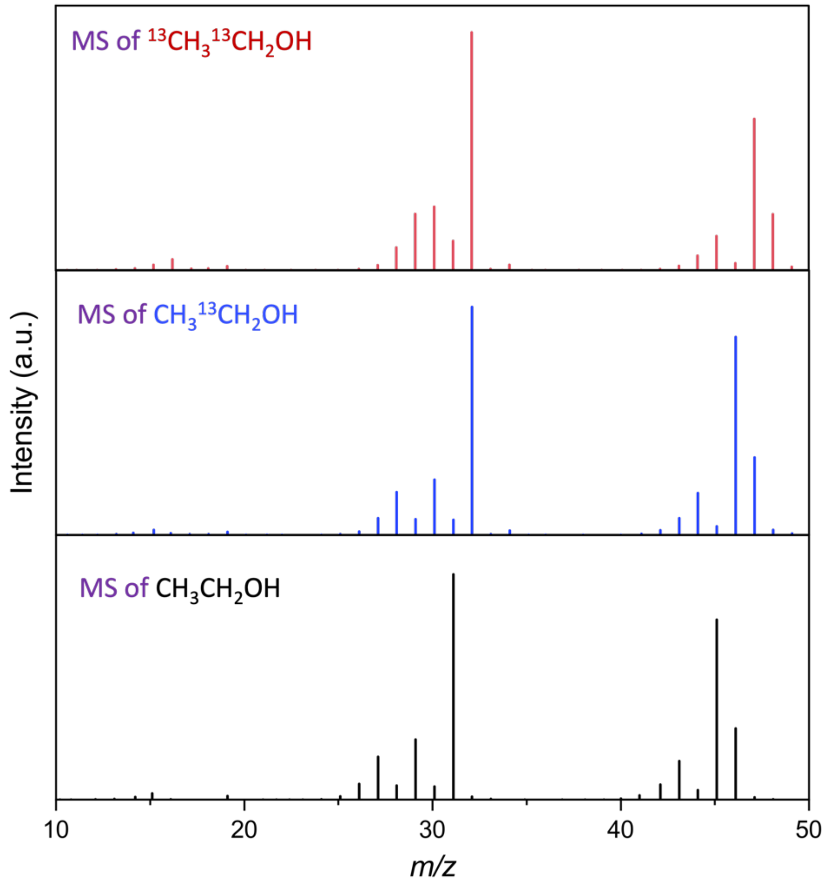

Figure S16. The MS spectra of the $^{13}CH_3{}^{13}CH_2OH$, $CH_3{}^{13}CH_2OH$ and CH_3CH_2OH.

The single-isotope labeled $CH_3{}^{13}CH_2OH$ exhibits a mass shift effect of (M +1) versus the

non-isotope labeled C_2H_5OH and the relative intensity of molecular ion peaks and the fragment ion peaks are consistent with the non-isotope labeled C_2H_5OH. The double-isotope labeled $^{13}CH_3{}^{13}CH_2OH$ exhibits a mass shift effect of (M +2) versus the non-isotope labeled C_2H_5OH, while the relative intensity of molecular ion peaks and the fragment ion peaks are slightly different from the single-isotope labeled $CH_3{}^{13}CH_2OH$ and non-isotope labeled C_2H_5OH

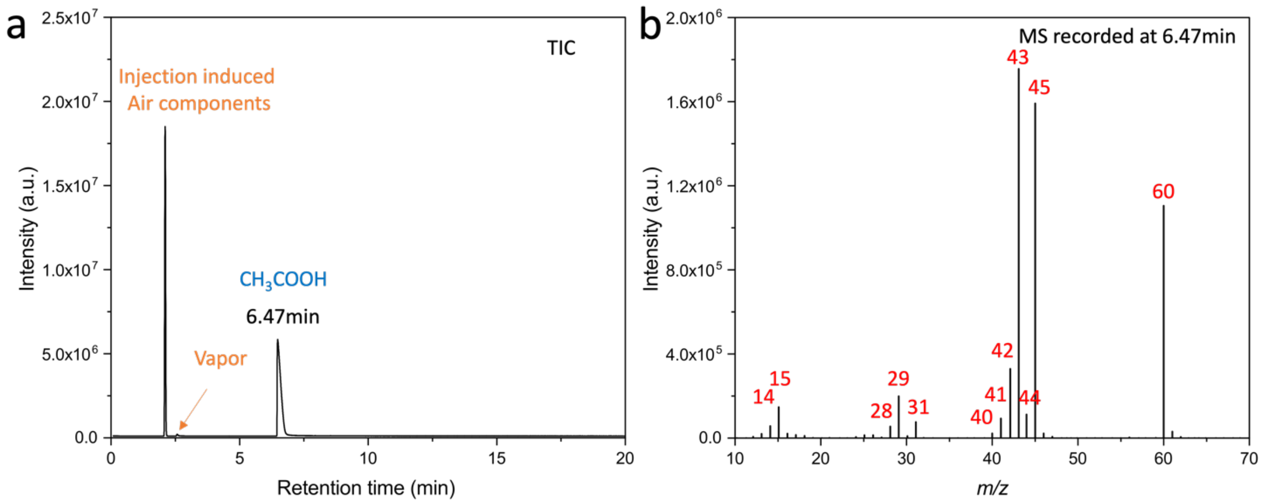

Figure S17. The TIC spectra of the CH_3COOH (a) and the corresponding MS spectra (b) recorded at 6.47 min in TIC.

The highest peak in MS spectra of CH_3COOH is the fragment ion of CH_3CO^+ since the dehydroxylation occurs easily during the ionization of acetic acid.

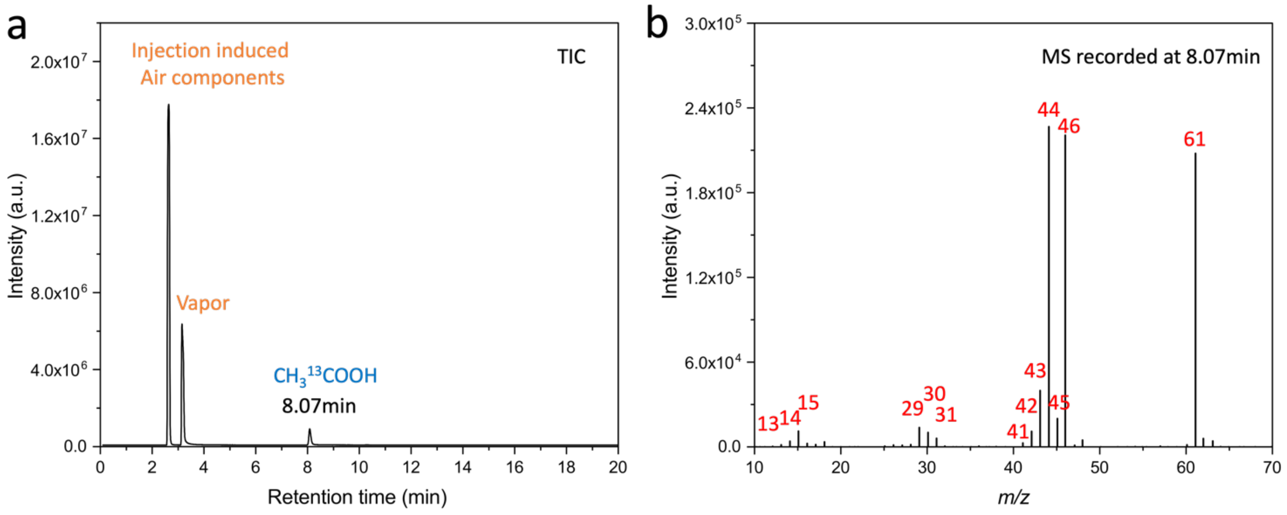

Figure S18. The TIC spectra of the $CH_3{}^{13}COOH$ (a) and the corresponding MS spectra (b) recorded at 8.07 min in TIC.

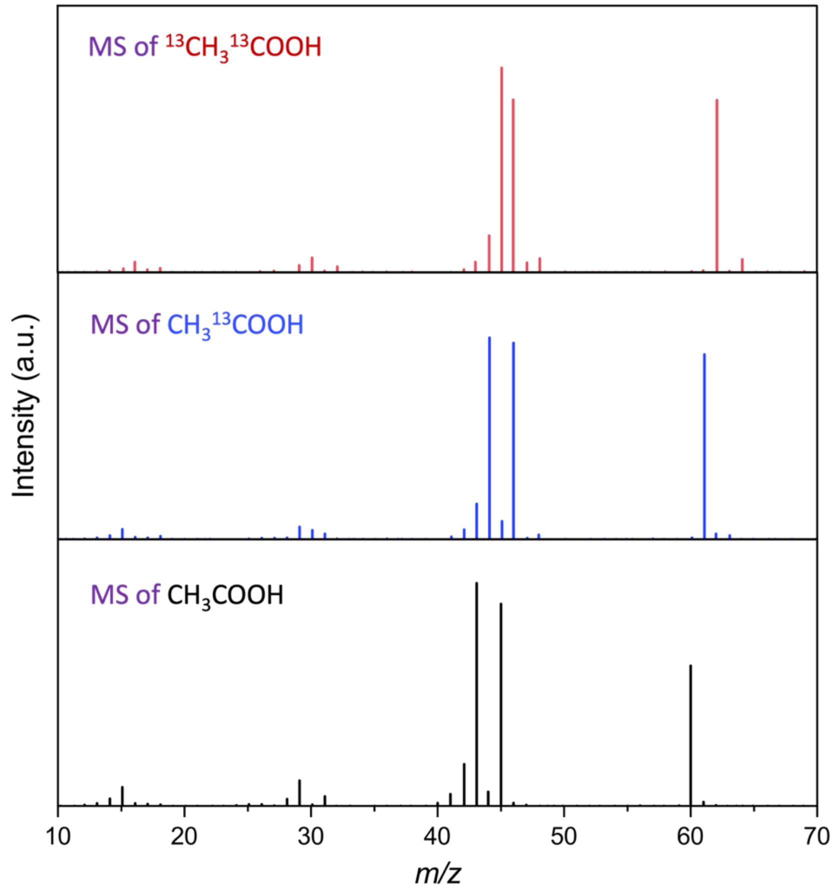

Figure S19. The MS spectra of the $^{13}CH_3{}^{13}CHOOH$, $CH_3{}^{13}COOH$ and CH_3COOH.

The single-isotope labeled $CH_3{}^{13}COOH$ exhibits a mass shift effect of (M +1) versus the non-isotope labeled CH_3COOH and the relative intensity of molecular ion peaks and the fragment ion peaks are consistent with the non-isotope labeled CH_3COOH. The double-

isotope labeled $^{13}CH_3{}^{13}COOH$ exhibits a mass shift effect of (M +2) versus the non-isotope labeled CH_3COOH, while the relative intensity of molecular ion peaks and the fragment ion peaks are slightly different from the single-isotope labeled $CH_3{}^{13}CH_2OH$ and non-isotope labeled C_2H_5OH due to the fragment ion peaks at m/z =45 and m/z =46 could be assigned to the $^{13}CH_3{}^{13}CO^+$ and $^{13}COOH^+$, respectively.

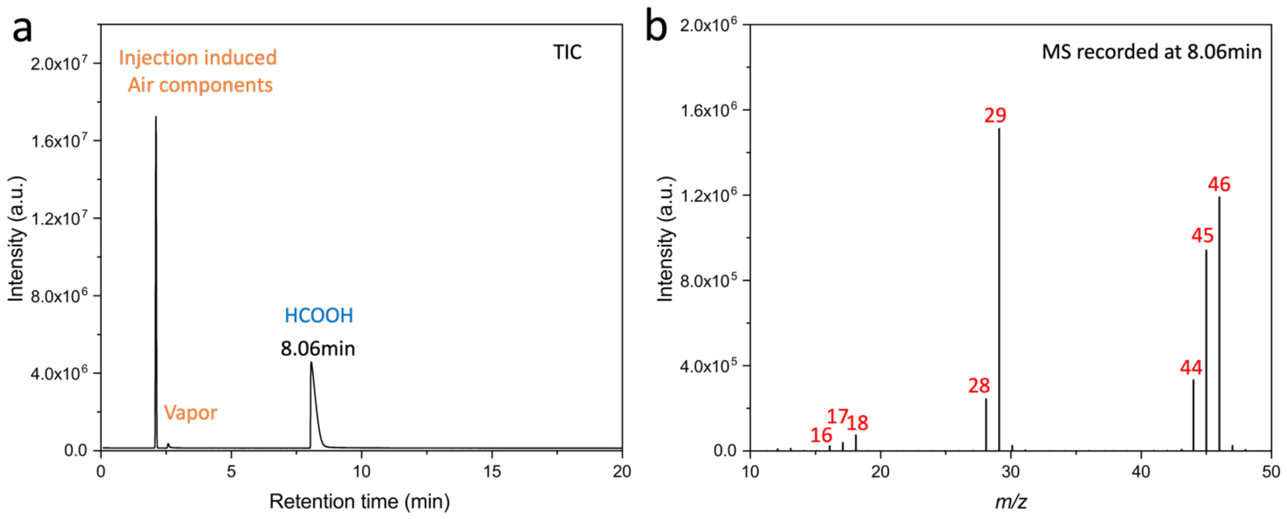

Figure S20. The TIC spectra of the HCOOH (a) and the corresponding MS spectra (b) recorded at 8.06 min in TIC.

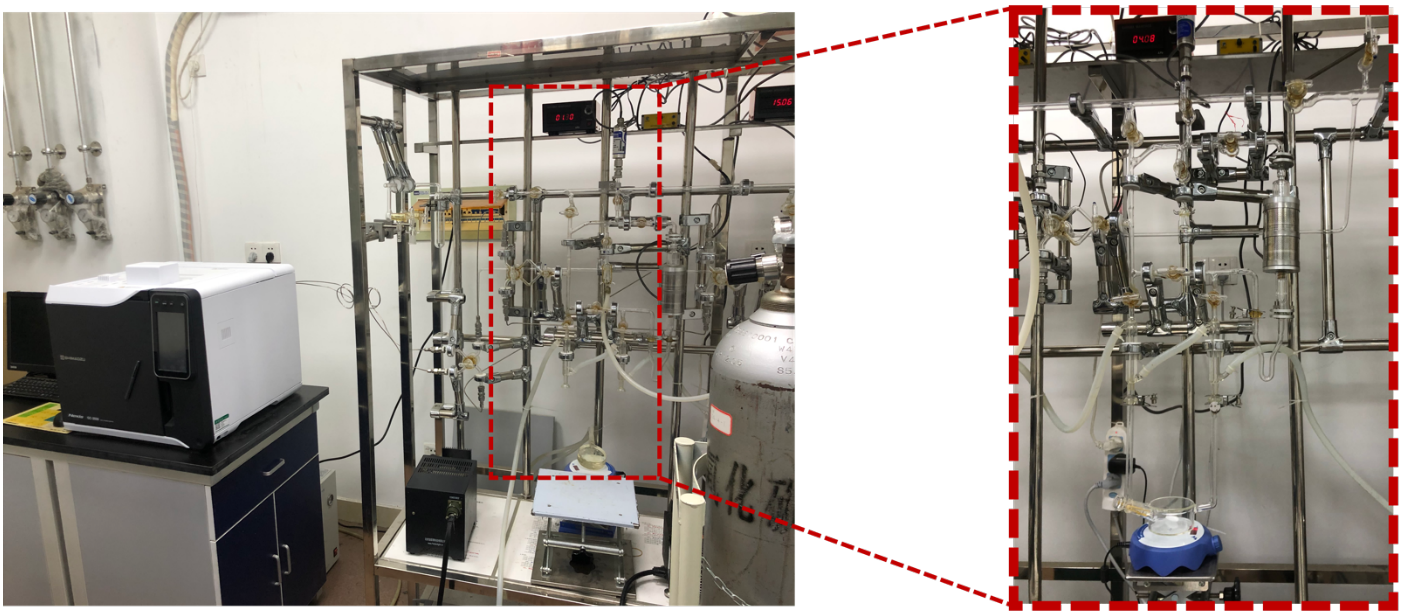

Figure S21. The closed gas circulation system for photocatalytic CO_2 reduction

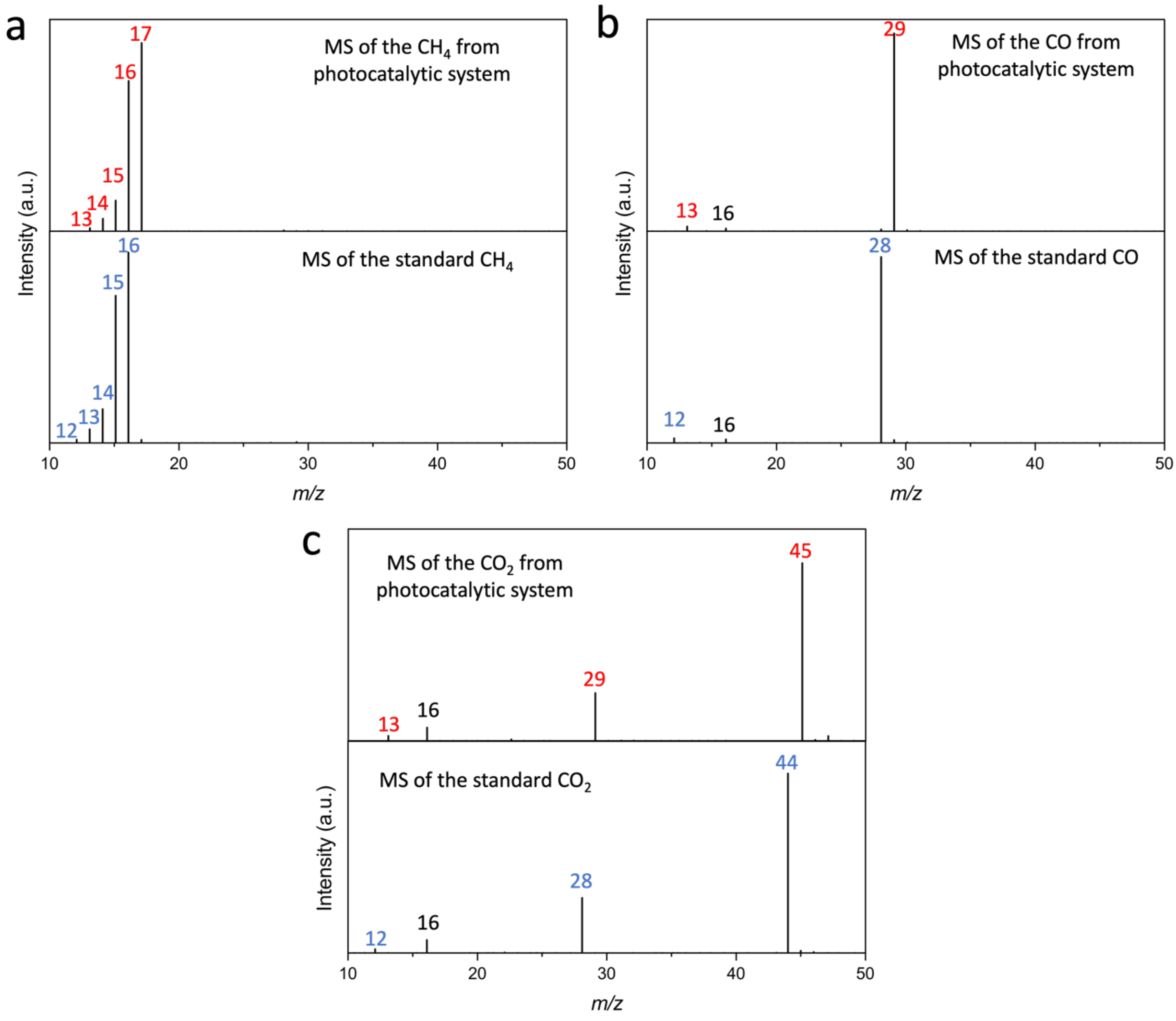

Figure S22. The MS spectra of CH_4, (a) CO (b) and CO_2 (c)from the photocatalytic system with Fe^{III} porphyrin complex as photocatalyst compared to the standard samples.

By comparison, it can be found that both the molecular ion peaks and fragment ion peaks of the reactant and products present a mass shift effect of (M +1) versus the non-isotope labeled sample, respectively.

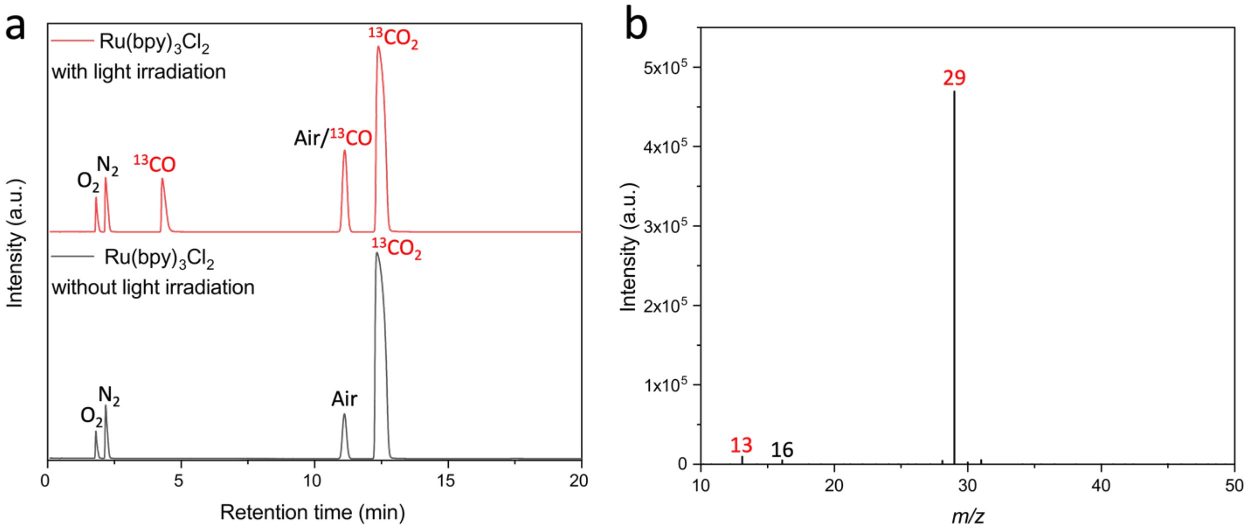

Figure S23. The TIC spectra of sample from the photocatalytic system with $Ru(bpy)_3Cl_2$ complex as photocatalyst before and after reaction (a) and the corresponding MS spectra of CO (b).

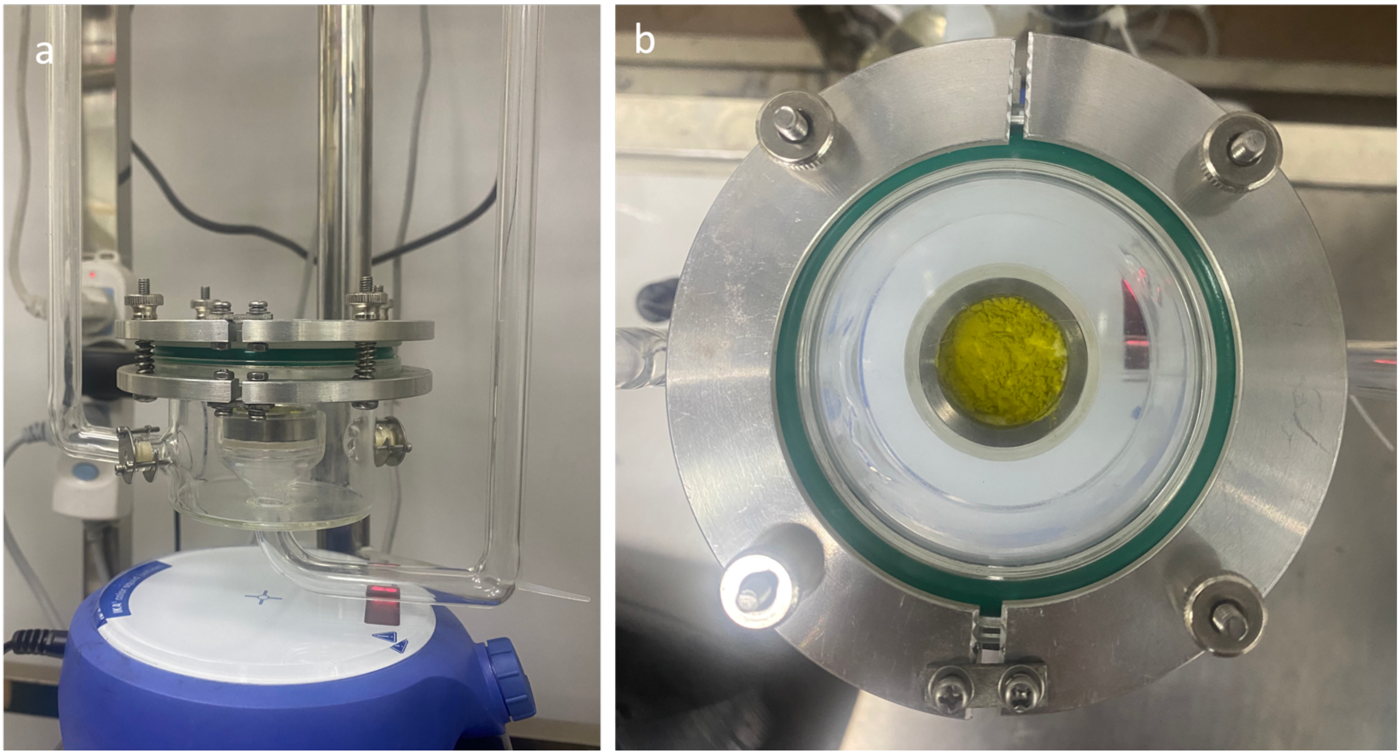

Figure S24. The side view (a) and top view (b) reaction cell for gas phase reaction

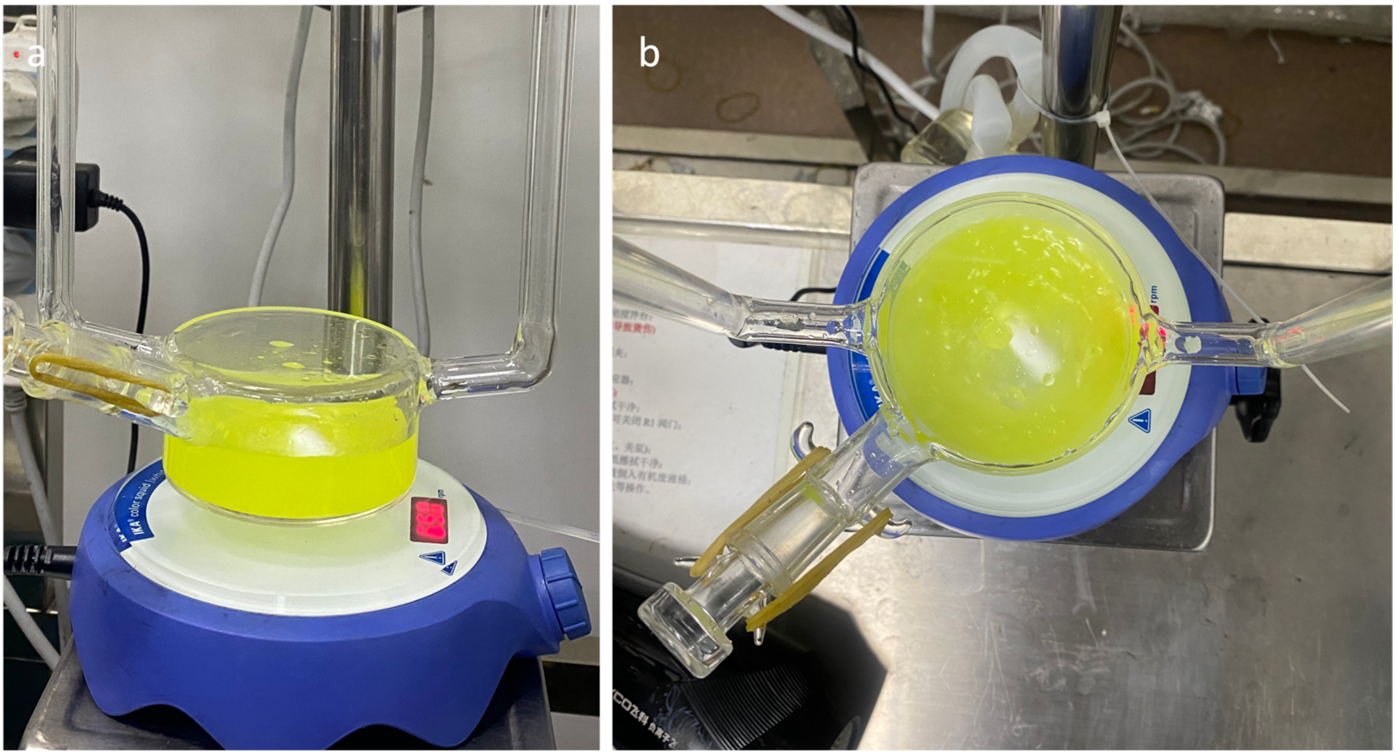

Figure S25. The side view (a) and top view (b) reaction cell for liquid phase reaction

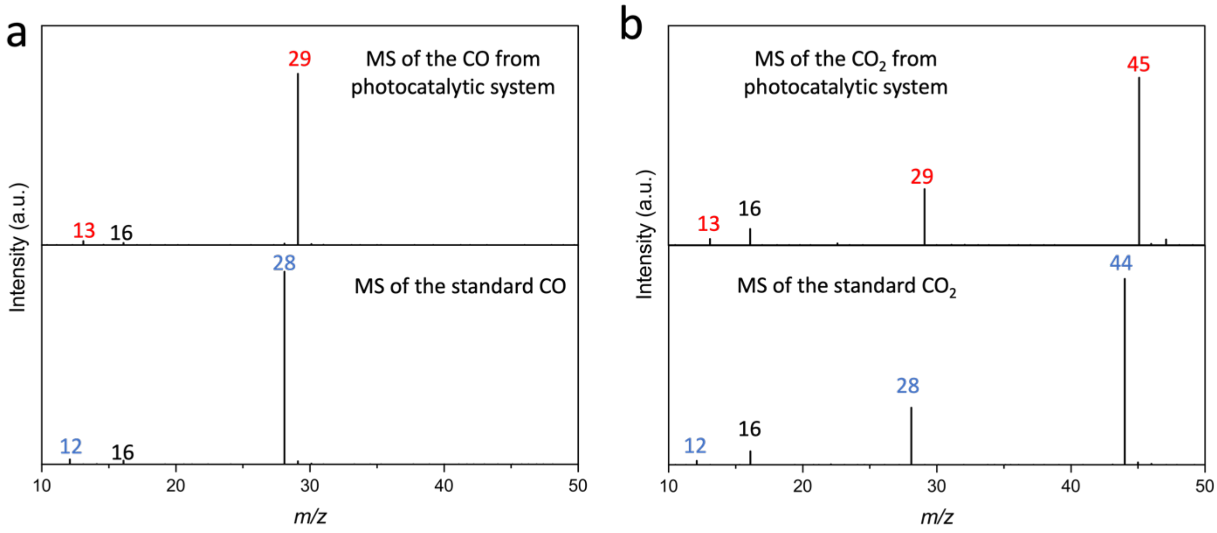

Figure S26. The MS spectra of CO (a) and CO_2 (b) from the liquid phase photocatalytic system with CPs as photocatalyst compared to the standard samples.

By comparison, it can be found that both the molecular ion peaks and fragment ion peaks of the reactant and products present a mass shift effect of (M +1) versus the non-isotope labeled sample, respectively.

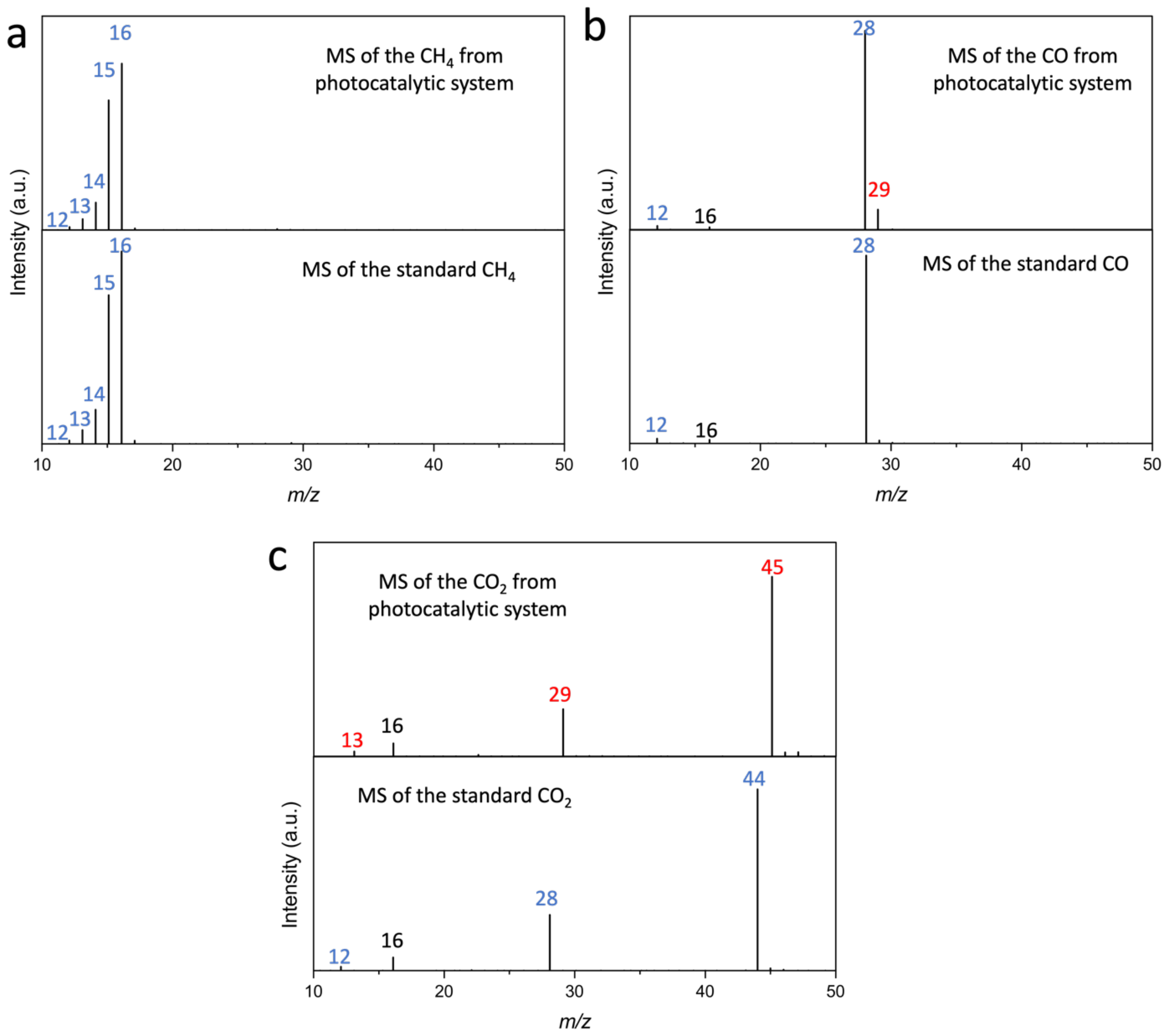

Figure S27. The MS spectra of CH_4, (a) CO (b) and CO_2 (c) from the gas phase photocatalytic system with CPs as photocatalyst compared to the standard samples.

By comparison, it can be found that the molecular ion peaks and fragment ion peaks of the reactant presents a mass shift effect of (M +1) versus the non-isotope labeled sample, while the molecular ion peaks and fragment ion peaks of product (CH_4) is the same as the non-isotope labeled sample. It shows that CH_4 is not obtained from the CO_2 photoreduction process. Although there is an additional peak located at m/z= 29 which could be assigned to the molecular ion peak of ^{13}CO, the mainly molecular ion peak and fragment ion peaks of CO_2 obtained from the photoreduction system is just like the standard CO. It shows that CO_2 is not mainly obtained from the CO_2 photoreduction process.
